# Multi-omics analyses demonstrate a critical role for EHMT1 methyltransferase in transcriptional repression during oogenesis

**DOI:** 10.1101/gr.277046.122

**Published:** 2023-01

**Authors:** Hannah Demond, Courtney W. Hanna, Juan Castillo-Fernandez, Fátima Santos, Evangelia K. Papachristou, Anne Segonds-Pichon, Kamal Kishore, Simon Andrews, Clive S. D'Santos, Gavin Kelsey

**Affiliations:** 1 Epigenetics Programme, Babraham Institute, Cambridge CB22 3AT, United Kingdom;; 2 Millennium Institute on Immunology and Immunotherapy, Laboratory of Integrative Biology (LIBi), Centro de Excelencia en Medicina Traslacional (CEMT), Scientific and Technological Bioresource Nucleus (BIOREN), Universidad de La Frontera, 4810296, Temuco, Chile;; 3 Centre for Trophoblast Research, University of Cambridge, Cambridge CB2 3EG, United Kingdom;; 4 Department of Physiology, Development, and Neuroscience, University of Cambridge, Cambridge CB2 3EG, United Kingdom;; 5 Cancer Research UK Cambridge Institute, Li Ka Shing Centre, University of Cambridge, Cambridge CB2 0RE, United Kingdom;; 6 Bioinformatics Group, Babraham Institute, Cambridge CB22 3AT, United Kingdom;; 7 Wellcome-MRC Institute of Metabolic Science–Metabolic Research Laboratories, Cambridge CB2 0QQ, United Kingdom

## Abstract

EHMT1 (also known as GLP) is a multifunctional protein, best known for its role as an H3K9me1 and H3K9me2 methyltransferase through its reportedly obligatory dimerization with EHMT2 (also known as G9A). Here, we investigated the role of EHMT1 in the oocyte in comparison to EHMT2 using oocyte-specific conditional knockout mouse models (*Ehmt2* cKO, *Ehmt1* cKO, *Ehmt1/2* cDKO), with ablation from the early phase of oocyte growth. Loss of EHMT1 in *Ehmt1* cKO and *Ehmt1/2* cDKO oocytes recapitulated meiotic defects observed in the *Ehmt2* cKO; however, there was a significant impairment in oocyte maturation and developmental competence in *Ehmt1* cKO and *Ehmt1/2* cDKO oocytes beyond that observed in the *Ehmt2* cKO. Consequently, loss of EHMT1 in oogenesis results, upon fertilization, in mid-gestation embryonic lethality. To identify H3K9 methylation and other meaningful biological changes in each mutant to explore the molecular functions of EHMT1 and EHMT2, we performed immunofluorescence imaging, multi-omics sequencing, and mass spectrometry (MS)–based proteome analyses in cKO oocytes. Although H3K9me1 was depleted only upon loss of EHMT1, H3K9me2 was decreased, and H3K9me2-enriched domains were eliminated equally upon loss of EHMT1 or EHMT2. Furthermore, there were more significant changes in the transcriptome, DNA methylome, and proteome in *Ehmt1/2* cDKO than *Ehmt2* cKO oocytes, with transcriptional derepression leading to increased protein abundance and local changes in genic DNA methylation in *Ehmt1/2* cDKO oocytes. Together, our findings suggest that EHMT1 contributes to local transcriptional repression in the oocyte, partially independent of EHMT2, and is critical for oogenesis and oocyte developmental competence.

The mammalian germline is the context for widespread epigenetic changes, in which the somatic epigenetic signature is erased and replaced by a germline signature that is distinct in sperm and oocytes. Before the onset of gametogenesis, primordial germ cells undergo extensive erasure of many epigenetic marks, such as DNA methylation (Guibert et al. 2012; Seisenberger et al. 2012) and histone-3 lysine-9 dimethylation (H3K9me2) (Seki et al. 2005). In the oocyte, epigenetic marks are reset postnatally during oocyte growth, resulting in a unique DNA methylation and histone modification landscape (Hanna et al. 2018a). As the oocyte does not divide during these processes, the oocyte serves as an informative system to study epigenetic regulation. Importantly, resetting of epigenetic marks is essential to support successful oogenesis, the oocyte-to-embryo transition, and subsequent embryonic development (e.g., Kaneda et al. 2004; Andreu-Vieyra et al. 2010; Eymery et al. 2016; Kim et al. 2016; Xu et al. 2019).

EHMT1 (also known as GLP) and EHMT2 (also known as G9A) are best known as histone methyltransferases, although they modify nonhistone targets as well (Rathert et al. 2008). They preferentially function as heterodimers in vivo and comprise the main H3K9 mono- and di-methyltransferases in euchromatin (Tachibana et al. 2001, 2005, 2008; Peters et al. 2003; Rice et al. 2003). Both proteins contain a SET domain, required for their catalytic activity as methyltransferases and heterodimer formation, as well as an ankyrin repeat domain that enables binding to H3K9me1 and H3K9me2 (Tachibana et al. 2001; Collins et al. 2008). EHMT1 and EHMT2 have been implicated in a number of cellular processes, including gene repression, higher-order chromatin structure, retrotransposon silencing, and DNA methylation, not all of which depend on their catalytic activity (Dong et al. 2008; Shinkai and Tachibana 2011; Bittencourt et al. 2014; Auclair et al. 2016; Au Yeung et al. 2019; Jiang et al. 2020). EHMT1 and EHMT2 have been suggested to be inter-dependent, as H3K9me1 and H3K9me2 levels are equally depleted in *Ehmt2* knockout (KO) and *Ehmt1* KO embryonic stem cells (ESCs) (Tachibana et al. 2005). Moreover, *Ehmt2* and *Ehmt1* mouse KOs show very similar phenotypes with embryos displaying loss of H3K9me2, growth retardation, and embryonic lethality between embryonic day (E) 8.5 and E12.5 (Tachibana et al. 2002, 2005). As such, studies often do not distinguish between EHMT1 and EHMT2, or solely consider EHMT2, resulting in limited knowledge of EHMT1 function. However, recent findings indicate that EHMT1 can act independently of EHMT2 early postfertilization, revealing a role for EHMT1 in targeting H3K27me2 to the paternal pronucleus in the zygote (Meng et al. 2020).

In the oocyte, loss of EHMT2 from the early growth phase after postnatal day 5 (as induced by the *Zp3*-Cre transgene driver) impairs maturation and meiosis, with consequences for preimplantation development, leading to partial embryonic lethality (Au Yeung et al. 2019). However, some embryos lacking oocyte-derived EHMT2 develop to term and result in healthy pups. The role of EHMT1 in the oocyte and its impact on embryo development remain unclear. To investigate the importance of EHMT1 in oogenesis and compare its function to EHMT2, we used oocyte cKO mice for *Ehmt2* and *Ehmt1*, as well as a *Ehmt1/2* cDKO. We use a multi-omics approach to evaluate changes in H3K9 methylation, gene expression, DNA methylation, and protein abundance in oocytes lacking EHMT1 and/or EHMT2. Our findings show that EHMT1 has a unique and essential function in the oocyte.

## Results

### EHMT1 and EHMT2 function during oocyte maturation and meiosis

**Figure 1.**
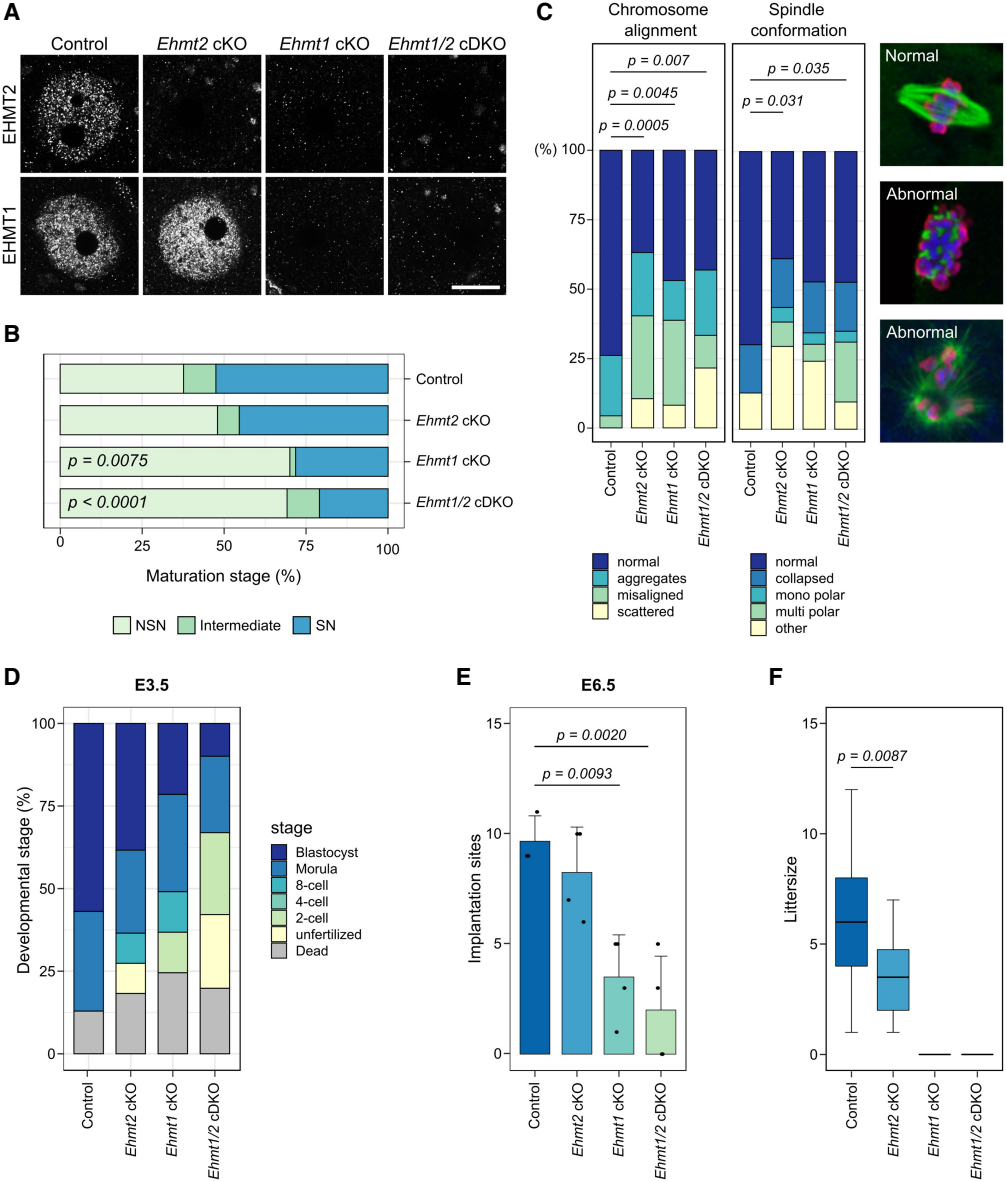
Developmental potential of *Ehmt2* cKO, *Ehmt1* cKO, and *Ehmt1/2* cDKO oocytes. (*A*) Representative images showing IF for EHMT2 and EHMT1 in NSN oocytes. Scale bar: 20 µm. (*B*) Stacked bar chart showing percentage of NSN, intermediate, and SN oocytes from mice aged 12 wk. Number of mice/oocytes: Control (Ctrl) = 5/247, *Ehmt2* cKO = 3/160, *Ehmt1* cKO = 2/116, *Ehmt1/2* cDKO = 3/188. Ctrl versus *Ehmt2* cKO: *P* = 0.7667; Ctrl versus *Ehmt1* cKO: *P* = 0.0075; Ctrl versus *Ehmt1/2* cDKO: *P* < 0.0001; *Ehmt2* cKO versus *Ehmt1/2* cDKO *P* = 0.0058. (*C*) Stacked bar charts showing percentage of chromosome misalignments and spindle abnormalities in MII oocytes. Examples of normal and abnormal spindles are shown in IF images. The spindle is stained with an anti-α-tubulin antibody (green), chromatin with DAPI (blue), and anti-pan-histone (red). Number of mice/MII oocytes: Ctrl = 5/46, *Ehmt2* cKO = 7/57, *Ehmt1* cKO = 4/49, *Ehmt1/2* cDKO = 6/51. (*D*) Stacked bar chart showing developmental stage (%) of embryos from cKO females mated with WT males and collected on E3.5. Number of mice/embryos: Ctrl = 5/30, *Ehmt2* cKO = 5/39, *Ehmt1* cKO = 6/35, *Ehmt1/2* cDKO = 6/29. Blastocysts: Ctrl versus Ehmt2 cKO *P* = 0.9939; Ctrl versus *Ehmt1* cKO *P* = 0.0761, Ctrl versus *Ehmt1/2* cDKO *P* = 0.0073; one-cell stage: Ctrl versus *Ehmt1/2* cDKO *P* = 0.0406. (*E*) Bar chart showing number of implantation sites scored at E6.5. Dots represent single mice. Ctrl versus *Ehmt1* cKO *P* = 0.0093; Ctrl versus *Ehmt1/2* cDKO *P* = 0.0020. (*F*) Boxplots showing average litter size of females with cKO oocytes mated with WT males. Number of mice: Ctrl = 9, *Ehmt2* cKO = 5, *Ehmt1* cKO = 3, *Ehmt1/2* cDKO = 3; number of litters: Ctrl = 60, *Ehmt2* cKO = 26, *Ehmt1* cKO = 0, *Ehmt1/2* cDKO = 0; Ctrl versus *Ehmt2* cKO: *P* = 0.0087.

To analyze the function of EHMT1 in oocytes and compare it to that of EHMT2, we generated three cKO models. Mice carrying floxed alleles for *Ehmt2* (*Ehmt2* cKO) (Sampath et al. 2007), *Ehmt1* (*Ehmt1* cKO) (Schaefer et al. 2009), or both *Ehmt2* and *Ehmt1* (*Ehmt1/2* cDKO) were crossed with a *Zp3*-Cre driver, which is expressed exclusively in growing oocytes after postnatal day 5 (Lan et al. 2004). During oocyte maturation in the ovary, the germinal vesicle (GV) oocyte undergoes a change in chromatin conformation from a nonsurrounded nucleolus (NSN) to a surrounded nucleolus (SN) stage that coincides with global transcriptional silencing (Zuccotti et al. 2005). In control animals, immunofluorescence (IF) analysis showed that EHMT1 and EHMT2 are detected throughout the nucleus of NSN oocytes but were no longer detectable by the mature SN stage (Supplemental Fig. S1A,B). In *Ehmt2* cKO oocytes, EHMT2 protein was lost in NSN oocytes, whereas EHMT1 remained readily detectable (Fig. 1A). In contrast, *Ehmt1* cKO oocytes were depleted for both EHMT2 and EHMT1, similar to *Ehmt1/2* cDKO oocytes (Fig. 1A), indicating that EHMT1 is required for EHMT2 stability but not vice versa, as was previously shown in mouse ESCs (Tachibana et al. 2005). This finding allows us to evaluate the role of EHMT1 independently of EHMT2 and whether EHMT1 is sufficient to maintain critical functions in the oocyte.

Loss of EHMT2 induced by the same *Zp3*-Cre driver has been shown to affect oocyte maturation and meiosis (Au Yeung et al. 2019). To assess whether loss of EHMT1 has additional effects on developmental capacity of the oocyte, we first analyzed the NSN-to-SN maturation rate by staining fully-grown GV oocytes with DAPI and staging them according to chromatin conformation. In *Ehmt1* cKO and *Ehmt1/2* cDKO oocytes from 12-wk-old females, the proportion of SN oocytes was significantly lower than that in *Ehmt2* cKO and control oocytes (Fig. 1B), indicating that loss of EHMT1 has a stronger effect on oocyte maturation than loss of EHMT2 alone.

To determine whether EHMT1 is required for meiosis, we analyzed spindle conformation and chromosome alignment of ovulated metaphase II (MII) oocytes after hormonal stimulation. All three cKO models showed an increase in abnormal chromatin configuration and spindle alignment compared with the controls (Fig. 1C). Various meiotic abnormalities were observed, ranging from chromosomes that were located together but not aligned (“aggregates”), to chromosome alignments in which one or several chromosomes were misaligned, to chromosomes scattered throughout the nucleus (Fig. 1C; Supplemental Fig. S1C). Spindle abnormalities included collapsed, monopolar, and multipolar spindles. No significant differences were observed between the different cKO models. Taken together, the results show that loss of EHMT2 had mild effects on oocyte maturation compared with the significant impairment caused by loss of EHMT1, whereas effects on meiosis were similar.

### Loss of maternal EHMT1, but not EHMT2, results in prenatal developmental arrest

To assess whether loss of EHMT1 from growing oocytes also compromises their competence, we examined developmental progression after fertilization. Embryos were collected at E3.5 from cKO females naturally mated with wild-type (WT) C57Bl/6Babr males and scored according to developmental stage. The majority of embryos from control females had reached the blastocyst stage (Fig. 1D). In comparison, fewer embryos derived from cKO oocytes progressed to blastocysts, with the proportion of maternal *Ehmt1/2* cDKO blastocysts significantly reduced, and the proportion of dead and unfertilized oocytes in maternal *Ehmt1/2* cDKO embryos significantly increased.

To determine the stage of embryo arrest, embryos were collected from superovulated, naturally mated cKO females at E1.5 and cultured in vitro for 3 d until E4.5. Already at E1.5, a difference was observed, with all three cKO models having a higher proportion of unfertilized oocytes and one-cell embryos than the controls. The *Ehmt1/2* cDKO again showed the strongest phenotype, having significantly fewer two-cell embryos than the controls (Supplemental Fig. S2A). Fertilized embryos (one and two cell) were selected for in vitro culture: None of the embryos from *Ehmt1* cKO or *Ehmt1/2* cDKO oocytes developed to blastocysts, arresting at earlier stages (one- to four-cell stage) by E3.5 (Supplemental Fig. S2B,C). In contrast, 42.9% maternal *Ehmt2* cKO embryos developed at least to morulae by E3.5 (Supplemental Fig. S2B,C). However, this was significantly less than the controls, in which 91.1% of embryos reached morula or blastocyst stages by E3.5. These findings reveal that embryos derived from *Ehmt2* cKO oocytes show reduced survival through preimplantation development, whereas the preimplantation developmental competence of *Ehmt1* cKO and *Ehmt1/2* cDKO oocytes was severely impaired.

A small percentage of embryos from *Ehmt1* cKO oocytes did progress in vivo to blastocysts; therefore, we examined the implantation and development of embryos after natural mating. In line with findings above, there were significantly fewer implanted embryos at E6.5 from *Ehmt1* cKO and *Ehmt1/2* cDKO oocytes than from *Ehmt2* cKO and the controls (Fig. 1E). At E8.5, three maternal *Ehmt1* cKO and six maternal *Ehmt1/2* cDKO embryos were recovered: All were highly abnormal, with no clear tissue types or with only extraembryonic tissue (Supplemental Fig. S2D,E). In contrast, although some abnormalities (predominantly developmental delay, but also abnormal morphology) were observed among E8.5 embryos from the *Ehmt2 c*KO oocytes, most appeared normal (Supplemental Fig. S2D,E). By E12.5, 55% (11/25) of the embryos from *Ehmt2* cKO oocytes were grossly abnormal or showed developmental delay (Supplemental Fig. S2F,G). In *Ehmt1/2* cDKO females, with one exception, all the embryos had died and only resorption sites were observed (Supplemental Fig. S2F).

Finally, we analyzed the number of live pups born after mating cKO females with WT males: No pups were born to *Ehmt1* cKO or *Ehmt1/2* cDKO females, but *Ehmt2* cKO females did give birth to a mean of 3.46 healthy pups per litter, a significantly reduced litter size compared with that of the controls (Fig. 1F). These results confirm previous findings in showing that although loss of EHMT2 from growing oocytes affects developmental capacity of pre- and postimplantation embryos, some embryos develop normally, resulting in birth of healthy pups (Au Yeung et al. 2019). Our results show loss of EHMT1 in growing oocytes severely impairs embryonic development, and although a small proportion of embryos reach the blastocyst stage and can implant, they die in utero between E8.5 and E12.5.

### Differential effects of loss of EHMT1 and EHMT2 on H3K9 methylation

**Figure 2.**
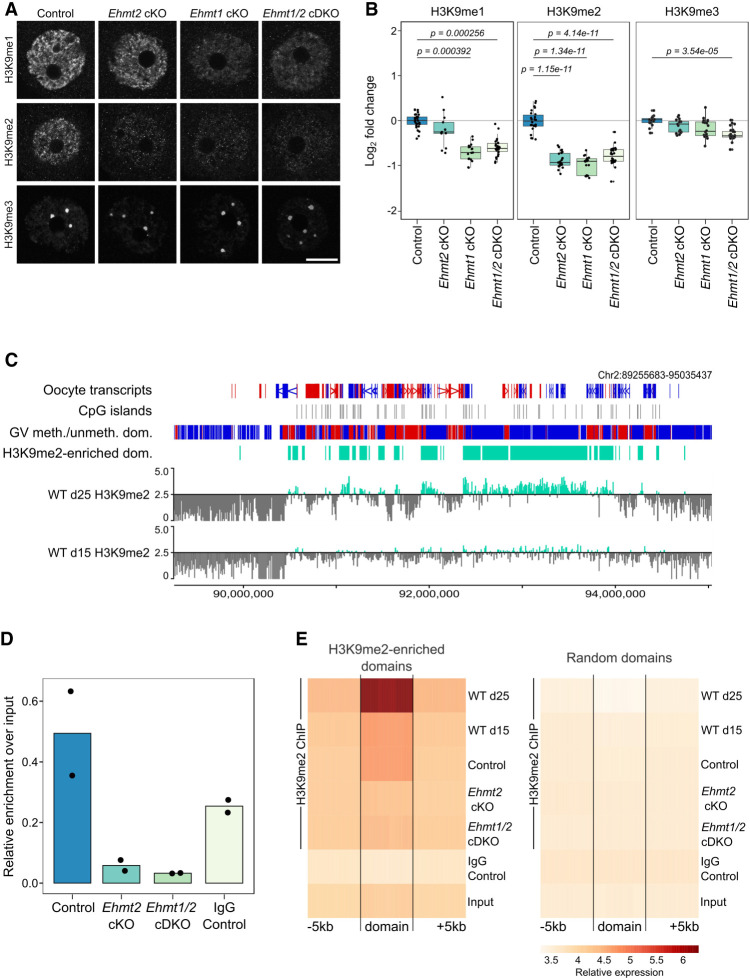
Analysis of H3K9 methylation in *Ehmt2* cKO, *Ehmt1* cKO, and *Ehmt1/2* cDKO oocytes. (*A*) Representative IF images of NSN oocytes for H3K9me1, H3K9me2, and H3K9me3 in control and *Ehmt2* cKO, *Ehmt1* cKO, and *Ehmt1/2* cDKO oocytes. Scale bar: 20 μm. (*B*) Boxplots of quantitation of IF images. Dots represent individual oocytes. Analysis is based on two to five mice per genotype and three to four replicate experiments for each antibody. (*C*) Genome screenshot showing H3K9me2 enrichment for 10-kb running windows in WT oocytes from mice aged 15 and 25 d. *Y*-axis scaling represents Log_2_RPKM centered around log_2_RPKM = 2.5. Annotation tracks highlight oocyte transcripts (forward in red, reverse in blue), CpG islands, oocyte DNA methylated (red) and unmethylated (blue) domains, and H3K9me2-enriched domains (defined as Log_2_RPKM > 2.5 in d25 GV oocytes). (*D*) Boxplots showing relative enrichment over input of H3K9me2 ChIP-seq libraries. Dots represent individual samples. (*E*) Probe trend plot showing loss of H3K9me2 enrichment in *Ehmt2* cKO and *Ehmt1/2* cDKO oocytes compared with WT and control oocytes in H3K9me2-enriched domains but not in random domains.

EHMT1 and EHMT2 are H3K9 methyltransferases required for establishment of H3K9me1 and H3K9me2 in euchromatin (Tachibana et al. 2001, 2002, 2005). Therefore, we examined H3K9 methylation in NSN-stage GV oocytes from cKO females (12 wk) by IF. We tested each antibody in three replicate experiments, each with oocytes from mice from a different litter. In total, we analyzed between 13 and 32 NSN oocytes per genotype per antibody (average, 22.7). Consistent with previous reports (Peters et al. 2003; Rice et al. 2003), H3K9me1 and H3K9me2 were localized mainly in euchromatic chromatin of NSN oocytes, whereas H3K9me3 was enriched in heterochromatic foci (Fig. 2A). The fluorescence intensity of H3K9me1 was significantly reduced in *Ehmt1* cKO and *Ehmt1/2* cDKO oocytes compared with the controls, but not in *Ehmt2* cKO oocytes; in contrast, H3K9me2 decreased significantly in all three cKOs (Fig. 2A,B). A small but significant loss of H3K9me3 was observed in *Ehmt1/2* cDKO oocytes (Fig. 2A,B). Rather than a direct effect, this may result from loss of H3K9me1, as the H3K9me3 methyltransferase SUV39H requires H3K9me1 as a substrate (Pinheiro et al. 2012). The loss of H3K9me2, but not H3K9me1 and H3K9me3, had previously been shown for *Ehmt2* cKO oocytes using different antibodies, showing the consistency of our IF experiments (Au Yeung et al. 2019).

With H3K9me1 and H3K9me2 affected by loss of EHMT2 and/or EHMT1, we sought to evaluate the genomic localization of these marks by ultra-low-input native ChIP-seq (ULI-nChIP-seq). Given the low-level, broad enrichment for H3K9me2 in GV oocytes, we performed the ChIP-seq experiments at two time points in WT GV oocytes from 15- and 25-d-old females to ensure we were obtaining robust, reproducible data sets. These time points represented an immature and mature GV oocyte state, respectively, with regards to accumulation of epigenetic marks, oocyte diameter, and NSN/SN state (Hiura et al. 2006; Hanna et al. 2018a). We were able to obtain quality ChIP-seq libraries for H3K9me2 (Fig. 2C), but not for H3K9me1. By quantifying 10-kb running windows, we observed a reproducible H3K9me2 enrichment between replicates from d15 and d25 WT oocytes, with d25 showing greater enrichment than d15 GV oocytes (Fig. 2C; Supplemental Fig. S3A). H3K9me2-enriched domains in d25 GV oocytes were defined by merging consecutive 10-kb windows with a Log_2_RPKM > 2.5 (34,192 or 12.5% of total 10-kb windows; 12,514 domains) (Fig. 2C; Supplemental Fig. S3B; Supplemental Table S1). To link H3K9me2 enrichment to transcription levels, we used published, deeply sequenced RNA-seq data spanning oocyte growth (Veselovska et al. 2015) to define CpG island (CGI) and non-CGI promoters of low (FPKM < 0.1), medium (FPKM 0.1–1.0), and high (FPKM > 1.0) expressed genes and then compared the overlap of these promoters with H3K9me2-enriched or random domains (Supplemental Fig. S3C). There was no difference in transcription level between genes localized to H3K9me2-enriched domains or random domains; thus, H3K9me2 enrichment does not appear linked to transcriptional repression in oocytes.

For further molecular analysis of EHMT1 function, we used the *Ehmt1/2* cDKO model, which had a slightly more severe phenotype than the *Ehmt1* cKO. As this small difference between *Ehmt1* cKO and *Ehmt1/2* cDKO oocytes might be caused by residual traces of EHMT2 in the *Ehmt1* cKO oocytes, using *Ehmt1/2* cDKO oocytes allowed us to make a clearer distinction between oocytes depleted of both EHMT2 and EHMT1 or of only EHMT2 (*Ehmt2* cKO). To assess the loss of H3K9me2 upon depletion of EHMT1 in the oocyte, we generated ChIP-seq libraries of GV oocytes from d25 *Ehmt2* cKO, *Ehmt1/2* cDKO, and littermate control females. Notably, H3K9me2 libraries from control oocytes were 4.94% of total chromatin, whereas H3K9me2 libraries from *Ehmt2* cKO and *Ehmt1/2* cDKO oocytes were only 0.58% and 0.33%, respectively (Fig. 2D), confirming that H3K9me2 is almost completely absent from cKO oocytes. Principal component analysis (PCA) of all biological replicates showed that *Ehmt2* cKO and *Ehmt1/2* cDKO H3K9me2 replicates clustered apart from control, d15 WT, and d25 WT H3K9me2 replicates (Supplemental Fig. S3D). When comparing enrichment across H3K9me2-enriched domains (±5 kb), *Ehmt2* cKO and *Ehmt1/2* cDKO showed significant loss of H3K9me2 compared with that of controls, with enrichment comparable to that in the IgG control and input (Fig. 2E). Furthermore, this effect was specific to H3K9me2-enriched domains, as there was no observable difference in enrichment between cKOs and controls across a set of random domains (Fig. 2E). There was a similar loss of H3K9me2 in *Ehmt2* cKO and *Ehmt1/2* cDKO oocytes, supporting the IF results that EHMT2 is predominantly required for H3K9me2 in oogenesis.

### Dysregulation of proteins associated with meiosis, fertilization, and oocyte function in *Ehmt1/2* cDKO oocytes

Beside their role as histone methyltransferases, EHMT1 and EHMT2 are known to methylate nonhistone proteins and, by doing so, can potentially modulate their function, localization, or stability. To assess whether loss of EHMT1 affects protein abundance in the oocyte, we performed low-input (200 oocytes) quantitative whole-proteome mass spectrometry isobaric labeling analysis of control, *Ehmt2* cKO, and *Ehmt1/2* cDKO GV oocytes. In total, 21,358 peptides were detected at FDR < 1% and 3182 proteins quantified (Supplemental Table S2). Gene Ontology (GO) analysis showed enrichment for processes involved in cellular localization and organization, as well as protein folding, metabolic processes, and fertilization (Supplemental Fig. 4A). Furthermore, among the top 50 most abundant proteins, we detected oocyte-specific proteins such as members of the subcortical maternal complex (PADI6, NLRP5, NLRP14, TLE6, KHDC3, NLRP4F), the zona pellucida (ZP1, ZP2, ZP3), as well as proteins known to be highly abundant in the oocyte (DNMT1, UHRF1).

**Figure 3.**
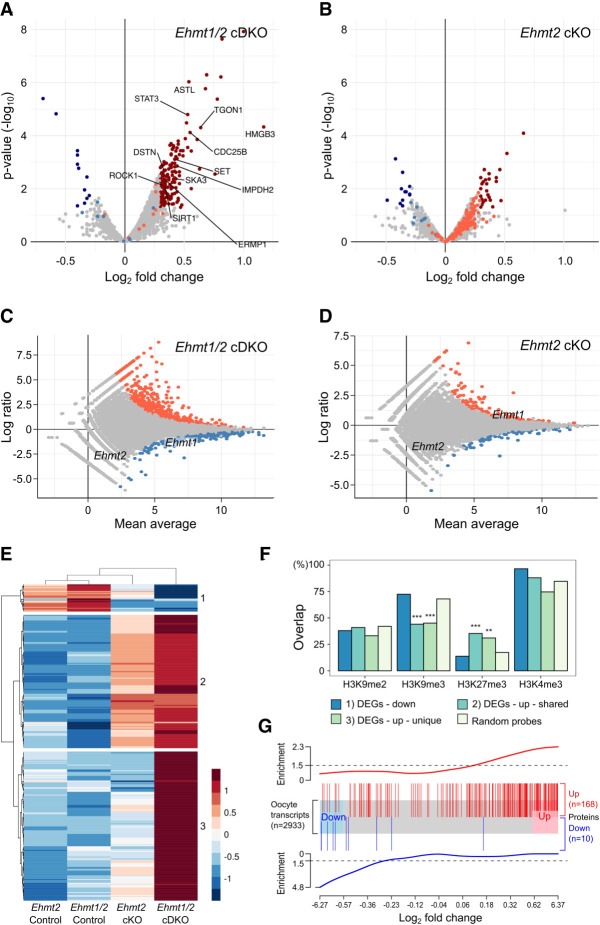
Proteome and transcriptome analysis of *Ehmt2* cKO and *Ehmt1/2* cDKO oocytes. (*A*) Volcano plot showing differential abundance of proteins in *Ehmt1/2* cDKO oocytes compared with the controls. Significantly changing proteins (*P* < 0.05 and Log_2_FC > 0.3) are highlighted in dark blue (decreased abundance) or dark red (increased abundance). Light blue and light red dots indicate proteins changing significantly in *Ehmt2* cKO oocytes. Proteins changing significantly and with oocyte function of interest are labeled. (*B*) Volcano plot showing differential abundance of proteins in *Ehmt2* cKO oocytes compared with the controls. Significantly changing proteins (*P* < 0.05 and Log_2_FC > 0.3) are highlighted in dark blue (decreased abundance) or dark red (increased abundance); light blue and light red dots indicate proteins changing significantly in cDKO oocytes. (*C*) MA plot showing Log_2_ fold changes in *Ehmt1/2* cDKO versus control oocytes (*y*-axis) over the mean expression level (*x*-axis). Differentially expressed genes (DEGs) are highlighted in red (up-regulated) and blue (down-regulated). (*D*) MA plot showing Log_2_ fold changes in *Ehmt2* cKO versus control oocytes (*y*-axis) over the mean expression level (*x*-axis). DEGs are highlighted in red (up-regulated) and blue (down-regulated). (*E*) Heatmap showing relative expression levels (Log_2_RPKM) of up- and down-regulated *Ehmt1/2* cDKO DEGs that overlap with *Ehmt2* cKO DEGs. (*F*) Bar chart showing proportion of DEGs overlapping with histone modifications. DEGs are split according to the clustering analysis of *E*. (**) *P* < 0.001, (***) *P* < 0.0001. (*G*) Plot showing link between changes in transcript and protein abundance. The *x*-axis shows Log_2_FC (control vs. *Ehmt1/2* cDKO) of genes present in both transcriptome and proteome data sets (*N* = 2933). Blue and red boxes represent down- and up-regulated transcripts, respectively, with a FC > 1.5 (Log_2_FC > 0.585). Vertical blue and red lines represent differentially abundant proteins (*P* < 0.05 and Log_2_FC > 0.3; 10 proteins not detected in the RNA-seq data are not represented). Enrichment scores, shown *above* and *below*, show that down-regulated proteins are enriched among down-regulated transcripts and up-regulated proteins enriched among up-regulated transcripts (Spearman's correlation *R* = 0.43; *P* = 2.2 × 10^−9^).

Comparing *Ehmt1/2* cDKO with control oocytes, we identified 187 proteins with a significant change in abundance (*P* < 0.05 and Log_2_FC > 0.3) (Fig. 3A; Supplemental Table S2). In contrast, there were only 38 differentially abundant proteins in *Ehmt2* cKO oocytes, of which 21 overlapped with those in *Ehmt1/2* cDKO oocytes (Fig. 3B; Supplemental Fig. 4B). The majority of changing proteins increased in abundance in both *Ehmt2* cKO (12 down, 26 up) and *Ehmt1/2* cDKO (12 down, 175 up) oocytes (Fig. 3A,B). Although few proteins were identified to have significant changes in abundance in *Ehmt2* cKO oocytes, many proteins changing in *Ehmt1/2* cDKO oocytes displayed the same directional trend in *Ehmt2* cKO oocytes (Fig. 3B).

We detected five known EHMT1/2 targets (ACIN1, DNMT1, DNMT3A, MTA1, and RUVBL2): None of these were significantly altered in cKO oocytes, but this is not entirely unexpected as protein methylation does not necessarily influence abundance but rather may affect activity. GO analysis did not detect any significant enrichment terms among the differentially abundant proteins. Even so, we identified several proteins for which change in abundance may be related to the observed oocyte phenotypes. Among these, two were meiotic factors according to the MGI Gene Ontology Browser (CDC25B and SIRT1), whereas literature research linked a further eight (STAT3, WDR1, TGON1, SET, IMPDH2, DSTN, SKA3, ROCK1) to meiosis in the oocyte (Supplemental Table S2). Furthermore, proteins involved in oocyte maturation (ERMP1) and fertilization (ASTL) showed significantly increased abundance, as well as the candidate oocyte transcriptional regulator HMGB3. Increased abundance of these proteins may contribute to the meiotic spindle abnormalities, impaired oocyte maturation, and poor fertilization rates observed in *Ehmt1/2* cKO oocytes. In line with observations in oocyte and embryo development, the more severe effect in *Ehmt1/2* cDKO oocytes suggests unique roles for EHMT1. The similar but lesser, nonsignificant changes in protein abundance observed in *Ehmt2* cKO oocytes suggests that loss of EHMT2 can be partially compensated by EHMT1.

### Transcriptome changes underlie differences in protein abundance in *Ehmt1/2* cDKO oocytes

To assess whether the proteome changes may be a consequence of transcriptional changes, we evaluated gene expression by RNA-seq of *Ehmt1/2* cDKO, *Ehmt2* cKO, and matched control GV oocytes. Each RNA-seq library was generated from the oocytes collected from a single mouse (70–200 GVs), which resulted in relatively small libraries with an average read number of 1.9 million; however, quality control analysis showed a high correlation between libraries (Supplemental Fig. S4C). Differential gene expression was determined by DESeq2 analysis followed by filtering for genes with Log_2_FC ≥ 1.5. In line with what was observed in the proteome, the vast majority of differentially expressed genes (DEGs) were up-regulated in the *Ehmt1/2* cDKO. Again, *Ehmt1/2* cDKO oocytes were more severely affected than *Ehmt2* cKO oocytes, with 330 DEGs (301 up, 29 down) (Fig. 3C; Supplemental Table S3), in contrast to 79 DEGs in *Ehmt2* cKO oocytes (64 up, 15 down) (Fig. 3D). Of the *Ehmt2* cKO DEGs, 51 overlapped with *Ehmt1/2* cDKO DEGs. We identified three clusters of DEGs: (1) down-regulated in both *Ehmt2* cKO and *Ehmt1/2* cDKO, (2) up-regulated in both genotypes, and (3) uniquely up-regulated in *Ehmt1/2* cDKO (Fig. 3E). GO analysis did not show significant category enrichments, but among the deregulated transcripts, we identified several transcription factors with a known function in the oocyte (*Prmt7*, *Etv5*), zygotic genome activation (*Zscan4d*), and embryo development (*Klf4*, *Hoxd1*, *Lmx1a*) (Supplemental Table S3). Furthermore, several genes important for oocyte maturation, meiosis, and fertilization were deregulated (*Atrx*, *Fgfr2*, *Prkcq*, *Ptgs2*, *Plac1*, *Mt1*), which may underlie some of the phenotypic effects we see. Consistent with the larger number and unique set of DEGs in the cDKO, the expression of long-terminal repeats (LTRs) of endogenous retroviral elements (ERVs) was up-regulated specifically in *Ehmt1/2* cDKO oocytes (Supplemental Fig. S5).

Using the DEG clusters, we assessed the overlap of DEGs with the distribution of histone modifications (Fig. 3F). There was no significant enrichment for any of the clusters with H3K9me2, further supporting the conclusion that H3K9me2 does not correlate with transcriptional repression in the oocyte nor does its loss explain the differences in severity between the *Ehmt2* cKO and *Ehmt1/2* cDKOs (Fig. 3F). Up-regulated DEGs (clusters 2 and 3) were low in H3K9me3, which in MII oocytes is localized in heterochromatic regions, such as LTRs (Wang et al. 2018). Conversely, up-regulated regions were enriched in H3K27me3, which is localized over untranscribed regions in the GV oocyte, but not in H3K4me3, which is also broadly localized over untranscribed regions but exclusive with H3K27me3 (Zheng et al. 2016; Hanna et al. 2018b). This finding suggests that up-regulated DEGs are loci that are transitioning from a repressed to active state in cKO oocytes.

Our proteome and transcriptome analyses showed a preferential up-regulation of protein and transcript abundance in *Ehmt1/2* cDKO oocytes. To assess whether changes in transcript expression may be causative for changes in protein abundance, we used gene set enrichment analysis (Fig. 3G). Indeed, proteins with increased abundance were enriched for transcriptionally up-regulated genes in *Ehmt1/2* cDKO oocytes, whereas proteins with decreased abundance were enriched for down-regulated genes, although this may not account for all the variation in protein abundance. This indicates that loss of EHMT1 results in deregulated gene expression, which in turn affects the abundance of the corresponding proteins. Hence, although only a minority of genes appears to be affected by loss of EHMT1, these transcriptional changes are likely to be of biological significance.

### Loss of EHMT1 results in local and distinct DNA methylation changes

**Figure 4.**
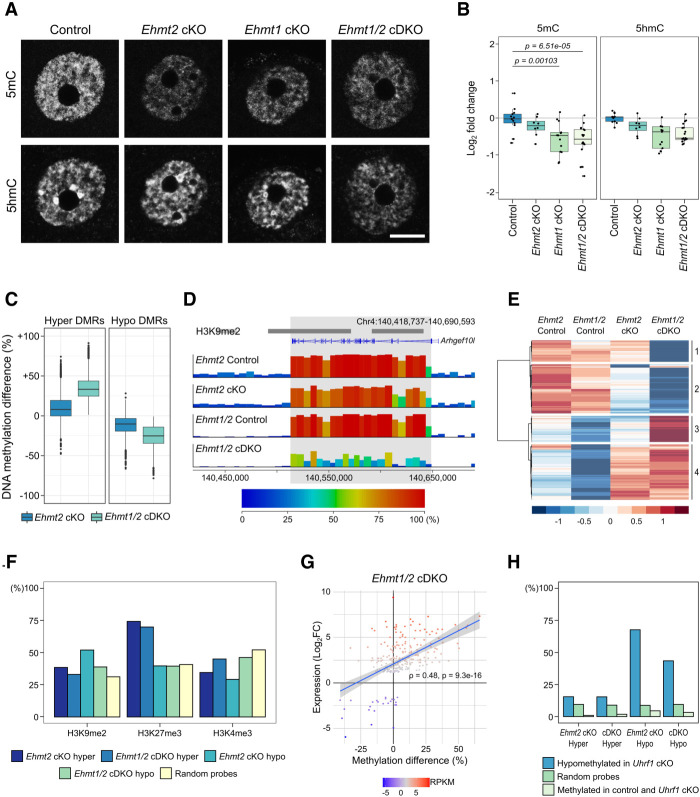
DNA methylation changes in *Ehmt2* cKO and *Ehmt1/2* cDKO oocytes. (*A*) Representative IF images of NSN oocytes showing 5mC and 5hmC in control, *Ehmt2* cKO, *Ehmt1* cKO, and *Ehmt1/2* cDKO oocytes. (*B*) Boxplots of quantitation of IF images. Dots represent individual oocytes. Analysis is based on two mice per genotype and two replicate experiments for each antibody. (*C*) Boxplot showing the methylation difference of *Ehmt1/2* cDKO DMRs (hyper and hypo) in controls versus *Ehmt2* cKO and controls versus *Ehmt1/2* cDKO oocytes. (*D*) Genome screenshot showing example of a region uniquely hypomethylated in *Ehmt1/2* cDKO oocytes spanning the *Arhgef10l* transcript. Annotation tracks show position of H3K9me2-enriched domains and oocyte transcripts. (*E*) Heatmap showing clustering analysis of *Ehmt1/2* cDKO hypo- and hypermethylated domains. (*F*) Bar chart showing percentage overlap of DMRs and random probes with H3K9me2, H3K37me3, and H3K4me3. H3K9me2: *Ehmt2* cKO hyper adj. *P* = 0.0136, *Ehmt2* cKO hypo *P* < 0.0001, *Ehmt1/2* cDKO hypo *P* < 0.0001; H3K27me3: *Ehmt2* cKO hyper *P* < 0.0001, *Ehmt1/2* cDKO hyper *P* < 0.0001; H3K4me3: all genotypes *P* < 0.0001. (*G*) Plot showing correlation between methylation changes (% methylation difference) and expression changes (Log_2_FC) of DEGs in *Ehmt1/2* cDKO oocytes. Relative expression levels (RPKM) of each gene are indicated by the color scale. Spearman's correlation is shown. (*H*) Bar chart showing percentage overlap of DMRs with 100-CpG windows that are hypomethylated in *Uhrf1* cKO oocytes (>20% methylation difference) (Maenohara et al. 2017), random 100-CpG windows, and 100-CpG windows that are highly methylated (>75%) in WT and *Uhrf1* cKO oocytes. *Ehmt2* cKO hyper adj. *P* = 0.0404; *Ehmt1/2* cDKO hyper, *Ehmt2* cKO hypo, and *Ehmt1/2* cDKO hyper *P* < 0.0001.

EHMT2-mediated H3K9me2 has been linked to DNA methylation (Tachibana et al. 2008; Zeng et al. 2019), although genome-wide analysis only detected local DNA methylation changes in *Ehmt2* cKO oocytes and *Ehmt2* KO embryos (Auclair et al. 2016; Au Yeung et al. 2019). To examine whether loss of EHMT1 affects DNA methylation establishment in the oocyte, we first analyzed global DNA methylation (5mC and 5hmC) by IF. We used a previously well-established method (Santos et al. 2013) and performed two replicate experiments with oocytes from different litters (average number of oocytes per genotype = 13). In NSN oocytes, 5mC was observed throughout the nucleus in both euchromatic and heterochromatic regions (Fig. 4A); 5hmC was enriched in heterochromatic foci but also present in euchromatin (Fig. 4A). No changes in localization of 5mC or 5hmC were observed in cKO oocytes. However, when assessing 5mC fluorescence intensity, there were significant decreases in the *Ehmt1* cKO and *Ehmt1/2* cDKO compared with the control oocytes, but not for *Ehmt2* cKO oocytes (Fig. 4B). No significant changes in 5hmC levels were observed in cKO oocytes, although 5hmC levels also appear reduced in the three cKO models compared with the controls. These data suggest that de novo DNA methylation may be impaired upon loss of EHMT1 but not EHMT2 in oocytes.

We then explored changes in DNA methylation in greater resolution by whole-genome bisulfite sequencing (BS-seq) of control, *Ehmt2* cKO, and *Ehmt1/2* cDKO GV oocytes. In contrast to decreased 5mC seen by IF, BS-seq did not show significant global changes in DNA methylation (Supplemental Fig. S6A). An explanation for this apparent discrepancy is that the genomic regions assessed by the two methods differ: IF signal reports both euchromatic and heterochromatic fractions of the genome, whereas BS-seq data are enriched in euchromatic and nonrepetitive regions that can be uniquely mapped and analyzed. These differences highlight the value of using both methods to understand genome-wide patterns of DNA methylation and may explain previous contradictory findings (Au Yeung et al. 2019; Zeng et al. 2019).

Although global DNA methylation levels as detected by BS-seq did not differ, local changes in DNA methylation were observed after binning the genome into consecutive windows of 100 CpG sites (100-CpG windows) for analysis. Differential methylation analysis identified 9187 DMRs in *Ehmt1/2* cDKO oocytes (4.88% of all analyzed 100-CpG windows), of which 4184 (45.5%) were hypermethylated (mean methylation difference: 35.1%) and 5003 (54.5%) hypomethylated (mean methylation difference 25.6%) (Supplemental Fig. S6B; Supplemental Table S4). Methylation was more affected in *Ehmt1/2* cDKO than in *Ehmt2* cKO oocytes, with greater than seven times more DMRs identified: There were 1252 DMRs in the *Ehmt2* cKO, of which 432 (34.5%) were hypermethylated (mean methylation difference: 39.4%) and 820 (65.5%) hypomethylated (mean methylation difference: 33.8%) (Supplemental Fig. S6C). The majority of *Ehmt2* cKO DMRs overlapped *Ehmt1/2* cDKO DMRs (Supplemental Fig. S6D). Furthermore, not only were there more DMRs, the methylation difference of these DMRs was greater in *Ehmt1/2* cDKO oocytes (Fig. 4C). This indicates that although both EHMT1 and EHMT2 are required for normal DNA methylation establishment in the oocyte, EHMT1 can partially compensate for loss of EHMT2, but it does not exclude the possibility that EHMT1 has an additional EHMT2-independent role in DNA methylation.

Between 21% and 38% of 100-CpG windows identified as DMRs cluster and form larger regions that, in both genotypes, can span genes (Fig. 4D; Supplemental Fig. S7A–C; Supplemental Table S4). In total, there were 707 hypomethylated and 869 hypermethylated domains in *Ehmt1/2* cDKO oocytes, with fewer in the *Ehmt2* cKO (140 hypomethylated and 66 hypermethylated domains). To see whether the additional changes in the *Ehmt1/2* cDKO are unique or whether similar (nonsignificant) trends are present in the *Ehmt2* cKO, we performed unsupervised cluster analysis (Fig. 4E), resulting in the identification of differentially methylated domains common to both cKOs (523 hypo, 590 hyper) or unique to the *Ehmt1/2* cDKO (229 hypo, 279 hyper) (Fig. 4D,E). This indicates that the majority of effects seen in the *Ehmt1/2* cDKO are also present in the *Ehmt2* cKO, although to a lower, often nonsignificant, magnitude. Therefore, there appears to be a potentially unique role of EHMT1 in oocyte DNA methylation, although this is likely to be minor compared with its function compensatory to loss of EHMT2.

### DNA methylation changes correlate with transcriptional changes and UHRF1-mediated methylation

To investigate possible underlying mechanisms, we analyzed the regions of the genome affected by DNA methylation changes. Although hypermethylated DMRs show similar overlap with genic and intergenic regions, hypomethylated DMRs are enriched in genic regions (Supplemental Fig. S6E). This is expected, as hypomethylated DMRs are found in regions that are methylated in control oocytes, and it is well established that DNA methylation localizes predominantly to transcribed genes in oocytes (Veselovska et al. 2015). To assess how DNA methylation changes correlate with underlying histone modifications, we tested the overlap of DMRs with H3K9me2, H3K27me3, and H3K4me3 (Fig. 4F). The majority of DMRs did not overlap regions with H3K9me2, and there was no clear distinction between hyper- and hypomethylated DMRs, indicating that most DNA methylation changes in cKO oocytes are unlikely to be a direct consequence of loss of H3K9me2. Hypermethylated DMRs were strongly enriched for H3K27me3 compared with hypomethylated DMRs. Importantly, this enrichment was not seen for H3K4me3, although both are enriched in regions of the genome lacking DNA methylation. Because H3K27me3 and DNA methylation are mutually exclusive, this result may indicate a localized redistribution of the two marks in cKOs.

As transcription is linked to the deposition of gene-body DNA methylation in the mouse oocyte, we analyzed the correlation between expression and DNA methylation changes. Consistently, expression changes of DEGs positively correlated with gene-body DNA methylation changes in *Ehmt1/2* cDKO oocytes (Fig. 4G). In contrast, when evaluating genes with differential methylation, the correlation was much weaker (Supplemental Fig. S6F). These trends were similar in *Ehmt2* cKO oocytes (Supplemental Fig. S6G,H). This shows that the transcriptional changes observed in *Ehmt1/2* cDKO oocytes impact the associated genic DNA methylation. However, this association does not explain most of the DNA methylation changes in *Ehmt1/2* cDKO oocytes; thus, other mechanisms likely underlie the majority of changes observed.

Because EHMT2 and EHMT1 can interact with UHRF1 (Kim et al. 2009; Ferry et al. 2017), we also investigated the overlap of DMRs with regions that are hypomethylated in *Uhrf1* cKO oocytes (Maenohara et al. 2017). We compared the overlap of *Ehmt2* cKO and *Ehmt1/2* cDKO hypomethylated DMRs with regions hypomethylated in *Uhrf1* cKO oocytes, random probes, and random probes that are highly methylated in control oocytes, as these are the regions most likely affected by loss of methylation. We observed that both *Ehmt2* cKO and *Ehmt1/2* cDKO hypomethylated DMRs strongly overlap with *Uhrf1* hypomethylated regions compared with random and random-methylated probes (Fig. 4H). These data suggest that loss of EHMT1 and EHMT2 may disturb a subset of UHRF1-mediated methylation. In summary, our analysis suggests that the genic DNA methylation gains seen in *Ehmt2* cKO and *Ehmt1/2* cDKO oocytes occur largely as a consequence to gene derepression, whereas losses of DNA methylation may be linked to impaired UHRF1-mediated de novo DNA methylation activity. These findings highlight that EHMT1 and EHMT2 are integral to several parallel molecular processes.

### Loss of EHMT1 and EHMT2 does not globally impair imprinting

Among hypermethylated loci in *Ehmt2* cKO and cDKO oocytes was the noncanonical imprinted gene *Gab1* (Supplemental Fig. S7A–C). As EHMT2 is required for noncanonical imprinting in embryos (Zeng et al. 2021), we systematically looked at noncanonical imprinted genes, which are within H3K27me3 domains in oocytes, and canonical imprinted genes, which are within DNA methylated domains. Of the seven noncanonical imprinted loci, *Gab1* was unique in showing increased DNA methylation (Supplemental Fig. S7D). *Gab1* also showed up-regulated expression in both *Ehmt2* cKO and cDKO oocytes (Supplemental Fig. S7E) consistent with loss of repressive chromatin leading to increased transcription and gain of DNA methylation. *Smoc1* and *Sfmbt2* were also up-regulated, but to a lesser magnitude, and not accompanied by altered DNA methylation (Supplemental Fig. S7E). Finally, examination of the maternal germline DMRs of canonical imprinted genes indicated the expected high levels of methylation (Supplemental Fig. S7D), which is in line with previous reports (Au Yeung et al. 2019).

## Discussion

**Figure 5.**
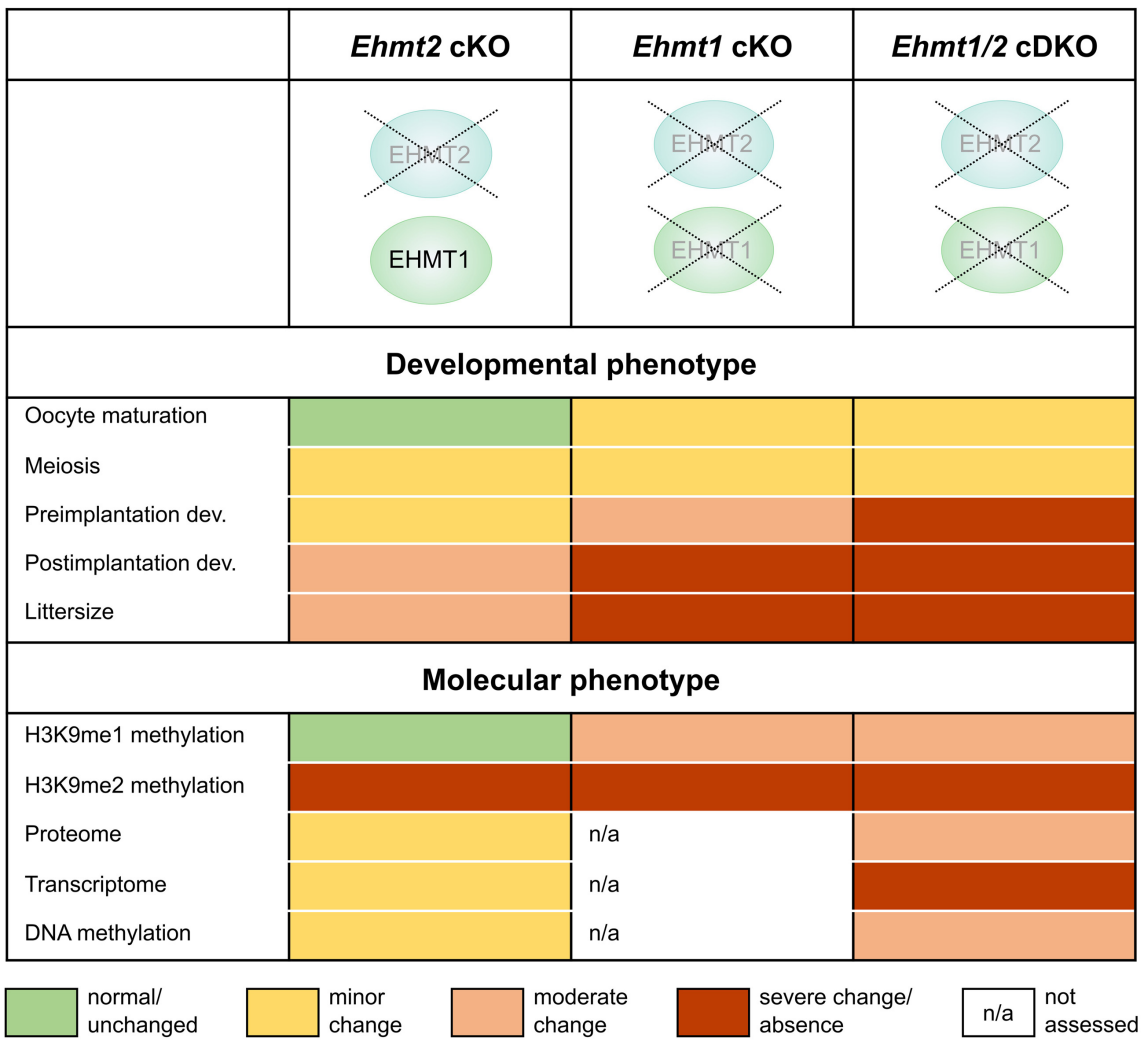
Summary of the developmental and molecular phenotypes of *Ehmt2* cKO, *Ehmt1* cKO, and *Ehmt1/2* cDKO oocytes, showing the distinct roles of EHMT1 and EHMT2 in the oocyte.

Our study shows that EHMT1 can partially compensate for loss of EHMT2 and additionally has a EHMT2-independent role in oogenesis (summarized in Fig. 5). We find that EHMT1 is required for oocyte maturation and developmental competence. Few embryos derived from *Ehmt1* cKO oocytes implant, and those that do die mid-gestation. In contrast, embryos from oocytes in which *Ehmt2* was ablated at the same time of oocyte growth are less affected, and some develop normally into healthy pups. This difference in severity is also reflected at a molecular level in oocytes, with a marked derepression of gene expression, which is correlated with increased protein abundance and gains in genic DNA methylation, and derepression of ERV LTRs. Notably, the differences between the genotypes are apparently independent of EHMT1's function as an H3K9me2 methyltransferase, as H3K9me2 was equally ablated in all cKOs as measured by IF and ChIP-seq. However, the loss of H3K9me1 only in the *Ehmt1* cKO and *Ehmt1/2* cDKO oocytes suggests that EHMT1's effect as a transcriptional repressor could be mediated in part through H3K9me1. Importantly, transcriptional changes do not explain all observed changes in DNA methylation with a marked overlap of regions that lose DNA methylation with those that are dependent on UHRF1, suggesting EHMT1 and EHMT2 may play a role in directing some UHRF1-facilitated DNA methylation.

To consider the molecular mechanisms underlying the observed phenotypes, it is important to appreciate that EHMT1 and EHMT2 are proteins with multiple functions. Although best known as histone methyltransferases, they can methylate and alter the function of nonhistone proteins (Shinkai and Tachibana 2011; Scheer and Zaph 2017). Nevertheless, the strong correlation we observed between changes in transcript and protein abundance in *Ehmt2* and *Ehmt1/2* cKO oocytes argues that the changes in protein abundance are likely attributable to altered genomic regulation rather than EHMT1/2 directly modulating protein stability.

Loss of EHMT1 and EHMT2 resulted in transcriptional up-regulation, supporting previous reports that EHMT1/2 act as repressors (Tachibana et al. 2002, 2005). It is unlikely that this can be attributed to loss of H3K9me2, because of the small number of genes that were up-regulated despite almost complete ablation of H3K9me2 and because of the significant transcriptional differences between the *Ehmt2* cKO and *Ehmt1/2* cDKO despite similar deficits in H3K9me2. Alternatively, the effects may be mediated indirectly by EHMT1/2 modulating activity of transcriptional repressors or through other chromatin changes, such as H3K9me1 or potentially H3K27me2. Further elucidation of whether changes in these repressive marks may be linked to transcriptional derepression remains a challenge owing to a lack of robust antibodies for ULI-ChIP-seq. We also observed up-regulation of some developmental transcription factors, suggesting that some gene expression changes reflect illegitimate expression of such factors in oocytes. The extensive transcriptional derepression in the *Ehmt1/2* cDKO is associated with impaired developmental capacity of the oocyte, resulting in decreased oocyte maturation, impaired fertilization, and abnormal embryo development.

EHMT1 and EHMT2 have been linked to DNA methylation in multiple studies. In the embryo, loss of EHMT2 leads to hypomethylation of a subset of CGI promoters (Auclair et al. 2016). In the oocyte, we and others do not see widespread hypomethylation (Au Yeung et al. 2019), but instead, local sites of hypo- and hypermethylation. A variety of mechanisms could underpin these changes. In the oocyte, de novo DNA methylation requires transcription (Kobayashi et al. 2012; Veselovska et al. 2015). The transcriptional changes in *Ehmt2* cKO and *Ehmt1/2* cDKO oocytes correlate with DNA methylation changes, indicating that up-regulated gene expression is responsible for some of the hypermethylation observed. The significant localization of *Ehmt2* cKO and *Ehmt1/2* cDKO derepressed genes and hypermethylated regions with H3K27me3 suggests that the loss of EHMT1/2 may impair the repressive chromatin landscape in a subset of regions in the oocyte. This could reflect its action in depositing H3K9me1/2, its activity as a transcriptional repressor, or its modulation of H3K27me2/3 levels, as EHMT1/2 have been reported to interact with and contribute to recruitment of PRC2 in mouse ESCs (Mozzetta et al. 2014). These mechanisms warrant further study, but possibly in other cell contexts because determining cause versus effect would be challenging in the oocyte.

A large subset of hypomethylated DMRs overlapped with regions that are hypomethylated in *Uhrf1* cKO oocytes (Maenohara et al. 2017). UHRF1 function has been associated with EHMT1 and EHMT2 in several ways, either by direct interaction (Kim et al. 2009) or by indirect recruitment through H3K9me2/3 or LIG1 (Rothbart et al. 2012, 2013; Ferry et al. 2017). However, it is not clear how these mechanisms apply in the oocyte because of the absence of DNA replication. Although UHRF1 appears to be required for some genomic DNA methylation, loss of DNMT1 has only minor effects, largely associated with its role in ensuring symmetric methylation of de novo methylated CpGs (Shirane et al. 2013; Maenohara et al. 2017). There are likely to be other mechanisms besides transcriptional regulation and UHRF1 interactions relevant in the *Ehmt2* cKO and *Ehmt1/2* cDKO oocytes. These include the possibility of a direct interaction of EHMT1 and EHMT2 with DNMT proteins; for example, EHMT1 and EHMT2 can dimethylate DNMT3A at lysine 44, and methylated DNMT3A can be bound by MPP8, which in turn binds automethylated EHMT1, resulting in a DNMT3A-MPP8-EHMT1 silencing complex (Chang et al. 2011).

In line with previous studies (Au Yeung et al. 2019), we find that EHMT2 is essential for H3K9me2 establishment in the oocyte, but not H3K9me1. Although H3K9me2 depends on the presence of EHMT2, the persistence of EHMT1 in the *Ehmt2* cKO was sufficient to establish H3K9me1. Differential requirements of EHMT1 and EHMT2 for H3K9me2 in GV oocytes were recently reported (Meng et al. 2022). Notably, loss of EHMT1 and EHMT2 did not result in a complete ablation of H3K9me1, indicating that other methyltransferases, such as PRDM3 and PRDM16, may be active in the oocyte. It remains unclear whether the decrease in H3K9me1 may be at least partially causative for the transcriptional and DNA methylation changes. Thus far, H3K9me1 has not been associated with transcriptional repression or de novo DNA methylation, but it is undercharacterized relative to H3K9me2/3 owing to a lack of validated, high-quality ChIP-grade antibodies.

While our paper was under review, a study was published in which *Ehmt2* was deleted before the onset of oocyte growth, driven by the oocyte-specific *Gdf9*-Cre transgene (Meng et al. 2022). Earlier ablation of *Ehmt2* led to a more severe oocyte developmental competence phenotype than we observed in our *Ehmt2* cKO or that reported by Au Yeung et al. (2019), but a phenotype with similarity to our *Ehmt1/2* cDKO oocytes. This raised the possibility that earlier loss of EHMT2 resulted in molecular defects similar to those caused by the later loss of EHMT1 (or combined absence of EHMT1/2 at the later stage). To explore this possibility, we compared the RNA-seq data from the three studies. To mitigate differences in RNA-seq library type, sequencing depth, and oocyte stage, we called DEGs separately in each data set using the same criteria. In brief, this analysis revealed a reasonable overlap between DEGs in our and the Au Yeung *Ehmt2* cKO, but a lower overlap with DEGs from the Meng *Ehmt2* cKO (Supplemental Fig. S8A,B). The overlap between the Meng DEGs and DEGs in our *Ehmt1/2* cDKO was also limited. Moreover, there was a strong bias toward up-regulated DEGs in our *Ehmt2* cKO and *Ehmt1/2* cDKO and the Au Yeung *Ehmt2* cKO (81%, 91%, 78%, respectively), but much less so in the Meng *Ehmt2* cKO (59%). Finally, Meng et al. (2022) identified down-regulation of *Ccnb3* as a major cause of the meiotic defects in their *Ehmt2* cKO. We find that *Ccnb3* mRNA is down-regulated in both our *Ehmt2* cKO and *Ehmt1/2* cDKO oocytes (Supplemental Fig. S8C), although it was not identified as DEG with the cut-offs used in our analysis. However, the reduction in *Ccnb3* transcript level in our *Ehmt2* cKO is more profound than in the *Ehmt1/2* cDKO oocytes, suggesting that defective *Ccnb3* expression does not explain the more strongly impaired developmental competence of the cDKO oocytes. Altogether, these findings suggest that the severity of the Meng *Ehmt2* cKO indicates a critical function of EHMT2 very early in oocyte growth, whereas EHMT1 has a separate critical function later in oocyte growth.

Our results indicate that EHMT1 can compensate for loss of EHMT2 in the oocyte, but also has unique roles. EHMT1 and EHMT2 are thought to function as heterodimers in vivo. Because EHMT2 is unstable on its own, it is technically challenging to create a model with intact EHMT2 in the absence of EHMT1. When comparing transcription, protein, and DNA methylation data from *Ehmt2* cKO and *Ehmt1/2* cDKO oocytes, some genes, proteins, and genomic regions are progressively affected; others, only in *Ehmt1/2* cDKO oocytes. The possibility of a unique, EHMT2-independent role for EHMT1 is supported by a recent study that reported that EHMT1 interacts with PRC2 in zygotes to establish H3K27me2 in the paternal pronucleus (Meng et al. 2020). In our models, the unique function was especially apparent in the transcriptome, where genes with the greatest fold-change were unique to the *Ehmt1/2* cDKO. The up-regulation seen in most DEGs was reflected in up-regulation of protein abundance in *Ehmt1/2* cDKO oocytes, indicating that although only a relatively small proportion of genes is derepressed upon loss of EHMT1 and EHMT2, the transcriptional changes are likely to have a functional impact in the oocyte. Indeed, we saw changes in gene expression and protein abundance of several genes related to oocyte maturation and fertilization. But the elevated or inappropriate expression of genes and corresponding proteins may be more deleterious than down-regulation, as very few genes are likely to be haploinsufficient in oocytes.

Taken together, our results highlight EHMT1 as a multifunctional repressive protein required for the appropriate establishment of the oocyte transcriptome, epigenome, and proteome. Consequently, EHMT1 is critical for the developmental capacity of the oocyte, independent of EHMT2.

## Methods

### Sample collections

All mice used in this study were bred and maintained in the Babraham Institute Biological Support Unit. Ambient temperature was ∼19°C–21°C; relative humidity, 52%. Lighting was provided on a 12-h light–12-h dark cycle including 15-min “dawn” and “dusk” periods of subdued lighting. After weaning, mice were transferred to individually ventilated cages with one to five mice per cage. Mice were fed CRM (P) VP diet (Special Diet Services) ad libitum and received seeds (e.g., sunflower, millet) at the time of cage cleaning as part of their environmental enrichment. All experimental procedures were performed under licenses issued by the Home Office (United Kingdom) in accordance with the Animals (Scientific Procedures) Act 1986 and were approved by the Animal Welfare and Ethical Review Body at the Babraham Institute.

Samples were collected from cKO mice carrying a *Zp3*-Cre driver in addition with floxed alleles for *Ehmt2* (Sampath et al. 2007), *Ehmt1* (Schaefer et al. 2009), or both. Oocytes and embryos were collected in M2 medium (Sigma-Aldrich M7167) unless stated otherwise. GV and MII oocytes for IF analysis were collected from adult mice aged ∼12 wk and fixed in 2% PFA for 15 min. MII oocytes were collected after superovulation. Oocytes used for ChIP-seq, BS-seq, and RNA-seq were collected from ovaries of 22- to 26-d-old mice. At this age, we did not observe a difference in NSN:SN ratio between the genotypes, therefore avoiding the possibility that any changes in H3K9me2, DNA methylation, or transcription would be caused by a shift between NSN:SN ratio. Oocytes were collected using a collagenase/trypsin digest. For ChIP-seq, 300 GV oocytes were collected in nuclear lysis buffer and pooled from two to four mice for each replicate. For BS-seq and RNA-seq, replicates were stored in RLT + buffer (Qiagen). Each replicate comprised all oocytes collected from one mouse (75–200 GV oocytes). For whole-proteome analysis oocytes were collected from ovaries of 22- to 26-d-old mice. To avoid contamination with proteins/peptides from the medium, oocytes were collected by manual dissection of ovaries in protein-free L15 medium (Thermo Fisher Scientific 31415029). About 200 GV oocytes were collected from two mice in parallel, washed three times in PBS with 1× complete protease inhibitor cocktail (Merck 11697498001).

Preimplantation embryos were collected after natural mating of control and cKO females with C57BL/6Babr WT males at E0.5 or E3.5. Implantation was determined by counting the number of implantation sites in uteri at E6.5 after timed mating. To score postimplantation development, embryos were collected after natural mating at E8.5 and E12.5. For embryo culture, female mice were superovulated and embryos dissected in M2 medium at E1.5 after natural mating with WT males. Fertilized embryos (one-cell and two-cell stage) were selected for culture in M16 medium (Sigma-Aldrich MR-016-D) under mineral oil (Sigma-Aldrich M8410) at 37°C and 5% CO_2_ for 3 d, and developmental progress was recorded each day.

### Analysis of maturation stage

GV oocytes from 12-wk-old females were stained with DAPI and scored according to their maturation stage. Absence of a ring around the nucleolus was counted as “NSN,” a partial ring as “intermediate.” and a full ring “SN.” Between 116 and 247 oocytes collected from several different females were analyzed per genotype (number of mice: control = 5, *Ehmt2* cKO = 3, *Ehmt1* cKO = 2, *Ehmt1/2* cDKO = 3).

### IF analysis

IF was performed after antibody staining as previously described (Santos et al. 2003). Primary antibodies are listed in Supplemental Table S5. MII oocytes were stained with antibodies against α-tubulin (spindle), pan-histone (chromatin), and DAPI (heterochromatic DNA). Between 46 and 57 MII oocytes from four to seven mice were analyzed per genotype. For H3K9me1, H3K9me2, H3K9me3, 5mC, and 5hmC, two or three replicate experiments were performed, each with 10–15 oocytes from different litters, to control for batch effects. Samples were analyzed on a Zeiss LSM780 confocal microscope (63× oil-immersion objective). Spindle conformation and chromosome alignment of MII oocytes were scored using the categories illustrated in Supplemental Figure S1C. For analysis of H3K9me and DNA methylation, Z-stacks of single optical sections were captured, and semiquantification of fluorescence intensity was performed using Volocity 6.3 (Improvision).

### LC-MS proteome analysis

Oocytes were lysed in 20 µL dissolution buffer containing 100 mM triethylammonium bicarbonate (Sigma-Aldrich T4708) and 0.1% sodium dodecyl sulfate (SDS), followed by water bath sonication and boiling for 5 min at 90°C. Proteins were reduced with tris-2-carboxyethyl phosphine (ΤCEP; Sigma-Aldrich) for 1 h at 60°C at a final concentration of 5 mM, followed by cysteine blocking for 10 min at room temperature using methyl methanethiosulfonate (MMTS; Sigma-Aldrich) at final concentration of 10 mM. Samples were digested overnight at 37°C with trypsin (Pierce 90058), and the next day peptides were labeled with TMT11plex reagents (0.4 mg per sample) according to the manufacturer's instructions (Thermo Fisher Scientific). To quench the reaction, 3 µL of 5% hydroxylamine (Thermo Fisher Scientific) was added for 15 min and samples combined and dried with centrifugal vacuum concentrator. The TMT mix was fractionated with reversed-phase spin columns at a high pH (Pierce 84868). Nine fractions were collected using different elution solutions in the range of 5%–50% ACN and were analyzed on a Dionex UltiMate 3000 UHPLC system coupled with the nano-ESI Fusion-Lumos (Thermo Fisher Scientific) mass spectrometer. Samples were loaded on the Acclaim PepMap 100, 100 μm × 2 cm C18, 5 μm, 100 Å trapping column with the ulPickUp injection method at loading flow rate 5 μL/min for 10 min. For peptide separation, the EASY-Spray analytical column 75 μm × 25 cm, C18, 2 μm, 100 Å column was used for multistep gradient elution. Full scans were performed in the Orbitrap in the range of 380–1500 m/z at 120 K resolution and peptides isolated in the quadrupole with isolation window 1.2 Th, HCD collision energy 38%, and resolution 50 K. Raw data were processed with the SequestHT search engine in Proteome Discoverer 2.1 software and searched against a UniProt database containing mouse reviewed entries. The parameters for the SequestHT node were precursor mass tolerance 20 ppm and fragment mass tolerance 0.02 Da; dynamic modifications were oxidation of M (+15.995 Da), deamidation of N, Q (+0.984 Da); and static modifications were TMT6plex at any N terminus, K (+229.163 Da), and methylthio at C (+45.988 Da). The consensus workflow included TMT signal-to-noise (S/N) calculation, and the level of confidence for peptide identifications was estimated using the percolator node with decoy database search. Strict FDR was set at *Q*-value < 0.01. For downstream data analysis, the R package qPLEXanalyzer was used (Papachristou et al. 2018).

### Preparation of sequencing libraries

ChIP-seq libraries were prepared using ULI-nChIP as previously described (Hanna et al. 2018b). Antibodies were added at a 250 ng/reaction for both anti-H3K9me2 (mouse monoclonal, Abcam ab1220) and anti-IgG (rabbit polyclonal, Diagenode EB-070-010). Library preparation was completed with a MicroPlex library preparation kit v2 (Diagenode) with Sanger eight-base indices for multiplexing. Relative enrichment over input was quantified using the library concentrations determined by Bioanalyzer high-sensitivity DNA analysis (Agilent). Low-input BS-seq libraries were prepared by postbisulfite adapter tagging as previously described (Hanna et al. 2018b). RNA-seq libraries were prepared as described (Hanna et al. 2019).

### Sequencing and data processing

Libraries were sequenced on Illumina MiSeq, HiSeq 2500, and NextSeq 500 systems. ChIP-seq libraries were sequenced to an average of 57 million paired-end reads of 75-bp read-length (Supplemental Table S6). BS-seq libraries were sequenced to an average of 16 million paired-end reads for *Ehmt2* cKO and 26 million reads for *Ehmt1/2* cDKO oocytes of 100–125 bp read-length. RNA-seq libraries were sequenced to an average single read number of 1.9 million for *Ehmt2* cKO and *Ehmt1/2* cDKO oocytes of 50-bp read-length. Raw FASTQ files were processed with Trim Galore! (https://www.bioinformatics.babraham.ac.uk/projects/trim_galore/) and then mapped to the mouse GRCm38 genome. Mapping of ChIP-seq data was performed using Bowtie 2 (Langmead and Salzberg 2012; https://bowtie-bio.sourceforge.net/bowtie2/index.shtml), RNA-seq data by HISAT2 v2.1.0 (http://daehwankimlab.github.io/hisat2/main/) guided by known splice sites, and BS-seq data with Bismark v0.19.0 (https://www.bioinformatics.babraham.ac.uk/projects/bismark/). Total sequencing read numbers and uniquely aligned read numbers are listed in Supplemental Table S6.

### Sequencing data analysis

Sequencing data analysis was conducted using SeqMonk (https://www.bioinformatics.babraham.ac.uk/projects/seqmonk/). For ChIP-seq analysis, 10-kb running windows (*N* = 272,566) were quantified as reads per kilobase per million (RPKM). Windows were filtered to exclude mapping artifacts, defined as RPKM > 6 in at least one replicate set of 10% input libraries (*N* = 408). H3K9me2 enrichment was defined as Log_2_RPKM > 2.5 in d25 GV oocytes (*N* = 34,192). A set of random windows was sampled from all 10-kb windows (*N* = 35,000). H3K9me2-enriched and random windows were then merged with adjacent windows within 10 kb, resulting in 12,154 H3K9me2-enriched domains and 26,077 random domains. Genic and intergenic regions were defined as overlapping or not overlapping oocyte transcripts, respectively, and promoters were defined as ±500 bp around transcription start sites of oocyte transcripts (Veselovska et al. 2015). CGIs were defined as previously described (Illingworth et al. 2008). Oocyte transcription levels were categorized into not expressed (FPKM < 0.1), low expressed (FPKM 0.1–1), and high expressed (FPKM > 1) using published data (Veselovska et al. 2015).

BS-seq data were analyzed using a tile-based approach of 100 CpGs for each consecutive genome window, ensuring equal CpG content in all windows. Methylation values were quantified using the bisulfite-sequencing pipeline quantification, which calculates per-base methylation percentages and averages these within each window. Filters were applied to ensure a minimum coverage of 10 or more observed cytosines per window. Only windows with this minimal coverage in all samples were taken into account, allowing assessment of 86.2% of 100-CpG windows (*N* = 188,433). Differentially methylated regions (DMRs) were defined by statistical comparison of DNA methylation levels for each 100-CpG window between control and *Ehmt2* cKO or control and *Ehmt1/2* cDKO oocytes using the edgeR function in SeqMonk. To assess overlap of DMRs with genomic features, CGI and oocyte gene annotations were used from Veselovska et al. (2015). DMRs were merged to form differentially methylated domains (DMDs). Hierarchical clustering analysis of DMDs was performed in R version 4.1.2 (2021-11-01) (R Core Team 2021) using Euclidean distance and Ward's agglomeration method as implemented by the package pheatmap (https://CRAN.R-project.org/package=pheatmap).

For analysis of RNA-seq data, the expression of oocyte genes (Veselovska et al. 2015) was quantified using log-transformed read count quantitation per million reads. Differential gene expression was analyzed using DESeq2 (Love et al. 2014) followed by filtering of genes with Log_2_FC > 1.5. When comparing DEGs between different data sets, libraries were size-normalized by down-sampling libraries to 1.9 million reads, representing the mean of the sample set with the least depth, before performing DESeq2. Clustering analysis of DEGs was performed in R using Euclidean distance and Ward's agglomeration method. Relative enrichment of differentially abundant proteins among DEGs was displayed using the barcode method from the Limma R package (Ritchie et al. 2015). The ranked list of expression changes only considered genes corresponding to proteins identified in the proteome. Spearman's rank correlation coefficient between DNA methylation difference and expression Log_2_FC was used to interrogate the relationship between DNA methylation and transcriptional changes. To quantify expression of ERVs, ERV annotations were downloaded from RepeatMasker (http://www.repeatmasker.org); ERVs within 2 kb of a gene were excluded to avoid spurious ERV calls from normal exonic gene expression, and then ERV counts were quantified as a percentage of total library counts. Publicly available ChIP-seq, RNA-seq, and BS-seq data accessed from the NCBI Gene Expresion Omnibus (GEO; https://www.ncbi.nlm.nih.gov/geo/) under accession numbers GSE93941, GSE112320, GSE153611, GSE97778, and DRA005849 were used to develop our studies.

### Statistical analysis

Statistical analysis was conducted in GraphPad Prism, R, and VassarStats. SN proportion was compared using two-way ANOVA (Stage × Genotype) with Šídák's multiple comparisons test. The proportion of embryo stages at E3.5, number of implantation sites at E6.5, and mean fluorescence intensity of confocal IF images were analyzed using one-way ANOVA with Tukey's post hoc test. Litter sizes were compared using a nested one-way ANOVA with mouse as nested factor. Overlap of DEGs with histone modifications was analyzed by comparing DEGs to random probes using a two-tailed Fisher's exact test. A *X*-square test was used to analyze meiosis abnormalities and the frequency of overlap of genic/intergenic regions with DMRs, histone modifications with DMRs, and UHRF1 hypomethylated regions with DMRs. *P*-value was adjusted using Bonferroni correction to control for multiple comparisons.

## Data access

The mass spectrometry proteomics data generated in this study have been submitted to the ProteomeXchange Consortium (http://www.proteomexchange.org/) via the PRIDE partner repository (Pérez-Riverol et al. 2019) under accession number PXD030265. All raw and processed sequencing data generated in this study have been submitted to the NCBI Gene Expression Omnibus (GEO; https://www.ncbi.nlm.nih.gov/geo/) under accession number GSE191026.

## Supplementary Material

Supplemental Material
