# Supplementary material for: Multi-omics analyses demonstrate a critical role for EHMT1 methyltransferase in transcriptional repression during oogenesis
Source: Genome Res. 2023 Jan;33(1):18–31. doi: 10.1101/gr.277046.122 (PMC9977154; doi:10.1101/gr.277046.122)
Supplement: Supplemental Material [file supp_33_1_18__DC1.html]

Multi-omics analyses demonstrate a critical role for EHMT1 methyltransferase in transcriptional repression during oogenesis — Multi-omics analyses demonstrate a critical role for EHMT1 methyltransferase in transcriptional repression during oogenesis — Supplemental Material 

# Multi-omics analyses demonstrate a critical role for EHMT1 methyltransferase in transcriptional repression during oogenesis

## Supplemental Material

- Supplemental\_Figures.docx
- Supplemental\_Table\_S1.xlsx
- Supplemental\_Table\_S2.xlsx
- Supplemental\_Table\_S3.xlsx
- Supplemental\_Table\_S4.xlsx
- Supplemental\_Table\_S5.xlsx
- Supplemental\_Table\_S6.xlsx
