# Supplementary material for: Multi-omics analyses demonstrate a critical role for EHMT1 methyltransferase in transcriptional repression during oogenesis
Source: Genome Res. 2023 Jan;33(1):18–31. doi: 10.1101/gr.277046.122 (PMC9977154; doi:10.1101/gr.277046.122)
Supplement: Supplemental Material [file supp_gr.277046.122_Supplemental_Figures.docx]

# Supplementary Figures


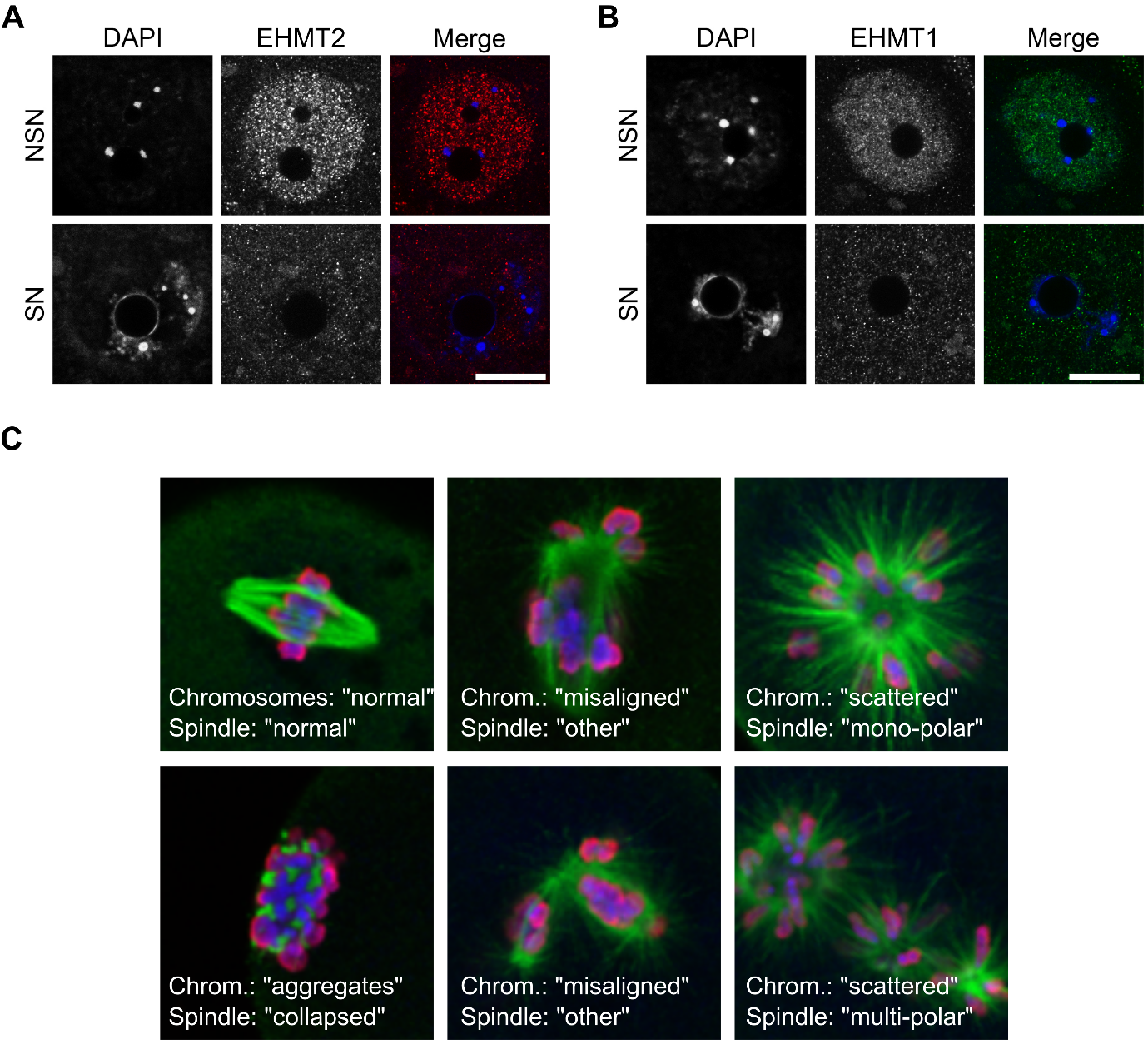


**Figure S1: EHMT1 and EHMT2 expression in the oocyte and their effects on meiosis**

**A, B)** Representative images showing DNA stained with DAPI, EHMT1 expression **(A)** and EHMT2 expression **(B)** revealed by IF in NSN and SN control oocytes. The scale bar represents 20µm. **C)** Example images showing meiotic abnormalities observed in MII oocytes. The spindle is stained with an anti-α-tubulin antibody (green) and the chromatin with DAPI (blue) and anti-pan-histone (red).

**
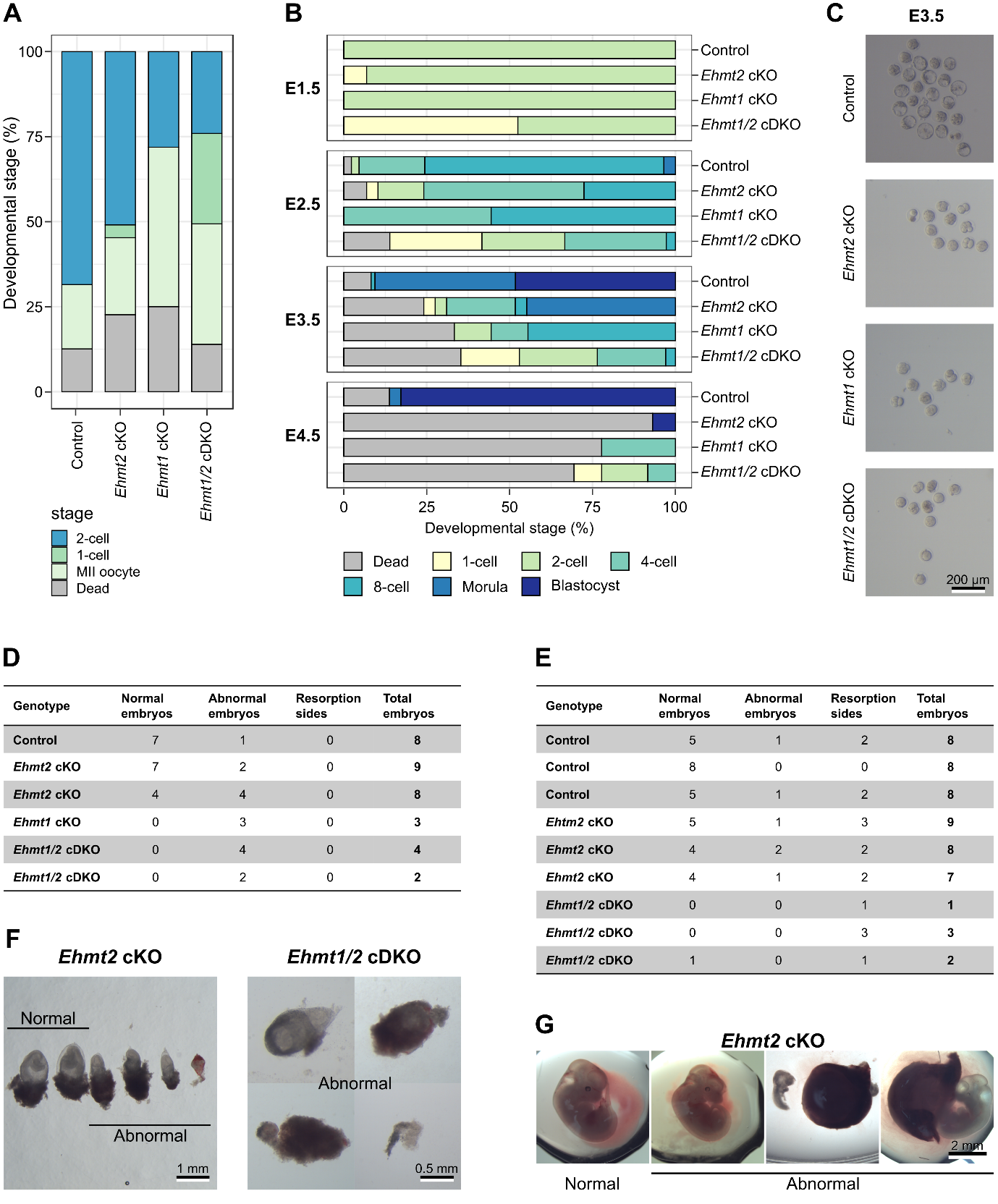
**

**Figure S2: *In vitro* preimplantation and *in vivo* postimplantation development of embryos lacking maternal EHMT2 and/or EHMT1.**

**A)** Stacked barchart showing developmental stage of embryos collected at E1.5 after superovulation and natural mating with C57Bl6/Babr WT males. One-way ANOVA with Tukey’s posthoc test: 2-cell stage Control vs *Ehmt1/2* cDKO *P = 0.0068***. **B)** Stacked barcharts showing developmental stage of embryos cultured *in vitro* after natural mating on E1.5, E2.5, E3.5 and E4.5. Number of litters/total number embryos: Control = 3/86, *Ehmt2* cKO = 3/29, *Ehmt1* cKO = 1/9, *Ehmt1/2* cDKO = 4/40. One-way ANOVA with Tukey’s posthoc test: morula stage Control vs *Ehmt2* cKO *P = 0.0006****. **C)** Images showing examples of embryos cultured in vitro on E3.5. **D, E)** Table showing embryo development at E8.5 **(D)** and E12.5 **(E)** after natural mating of conditional KO females with WT males. Each line represents one female mouse. Indicated are the number of normal embryos, abnormal embryos and resorption sides for each female. **F,G)** Representative images showing normal and abnormal embryos at E8.5 **(F)** and E12.5 **(G)**, corresponding to the tables in **D** and **E**.

**
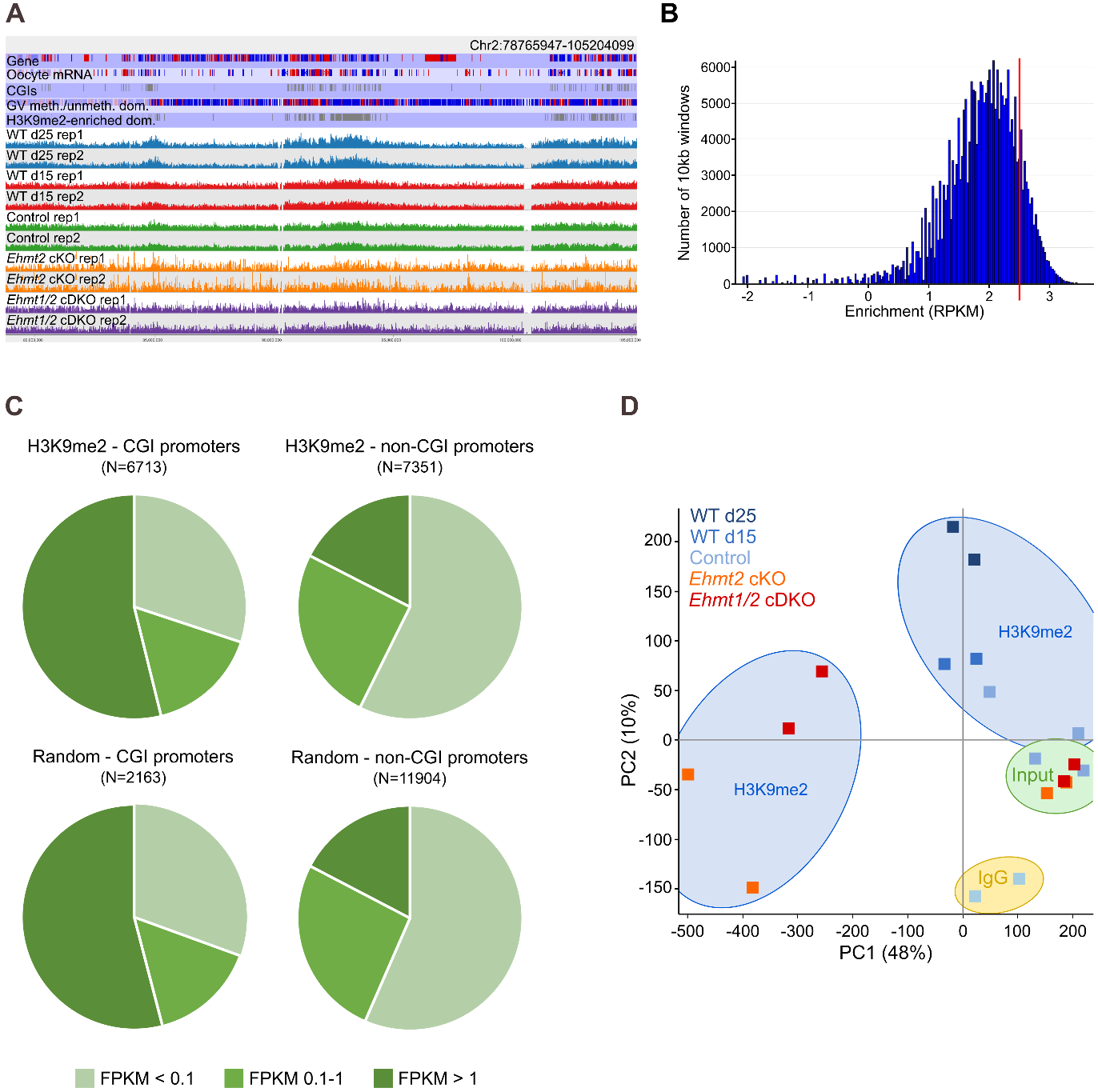
**

**Figure S3: ChIP-seq analysis showing H3K9me2 distribution in control, *Ehmt2* cKO and *Ehmt1/2* cDKO oocytes.**

**A)** Screenshot showing H3K9me2 enrichment and reproducibility of replicates. **B)** Histogram showing number of 10 kb windows with a certain enrichment level in d25 WT oocytes. The red line indicates the threshold set to define H3K9me2 enrichment (RPKM > 2.5). **C)** Pie charts showing overlap of H3K9me2 enriched and random probes with untranscribed (FPKM < 0.1), lowly transcribed (FPKM 0.1-1) and highly transcribed genes (FPKM > 1). **D)** PCA plot of H3K9me2 (blue shading), input (green shading) and IgG (yellow shading) ChIP-seq libraries, comparing WT (d15 and d25), control, *Ehmt2* cKO and *Ehmt1/2* cDKO samples.

**
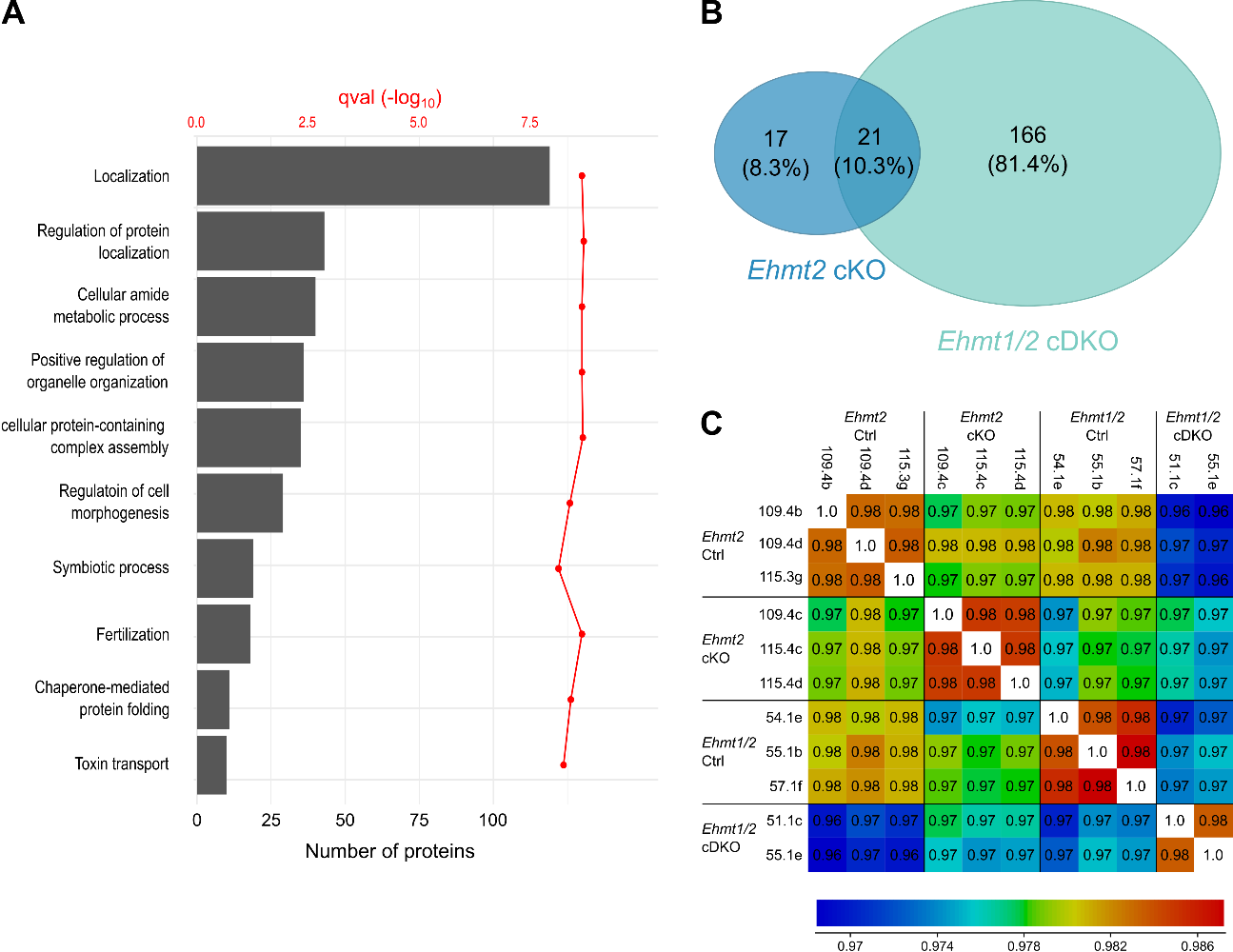
**

**Figure S4: Proteome and transcriptome analysis of *Ehmt2* cKO and *Ehmt1/2* cDKO oocytes with corresponding littermate controls.**

**A)** GO analysis of the oocyte proteome. **B)** Venn diagram of significant changing proteins in *Ehmt2* cKO and *Ehmt1/2* cDKO oocytes. **C)** Correlation matrix for *Ehmt2* cKO, *Ehmt1/2* cDKO and corresponding control GV oocyte RNA-seq libraries.


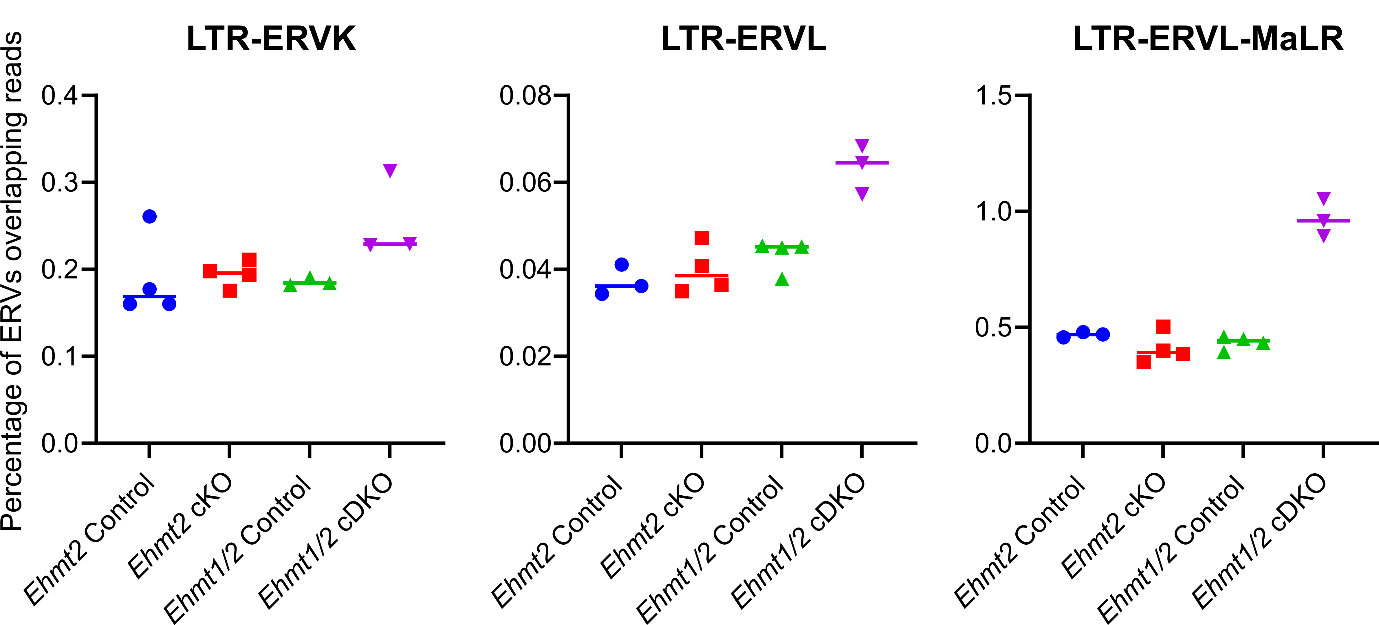


**Figure S5: Expression of ERV-LTRs in *Ehmt2* cKO and *Ehmt1/2* cDKO oocytes.**

Plots showing total expression levels of ERVs as a percentage of total library counts in individual RNA-seq datasets from *Ehmt2* cKO and *Ehmt1/2* cDKO oocytes. Expression of LTR-ERVL (Unpaired t-test: p=0.0023**) and LTR-ERVL-MaLR (p<0.0001****) was significantly up-regulated in *Ehmt1/2* cDKO oocytes, but not LTR-ERVK (p=0.0649). None were significantly different in *Ehmt2* cKO oocytes.


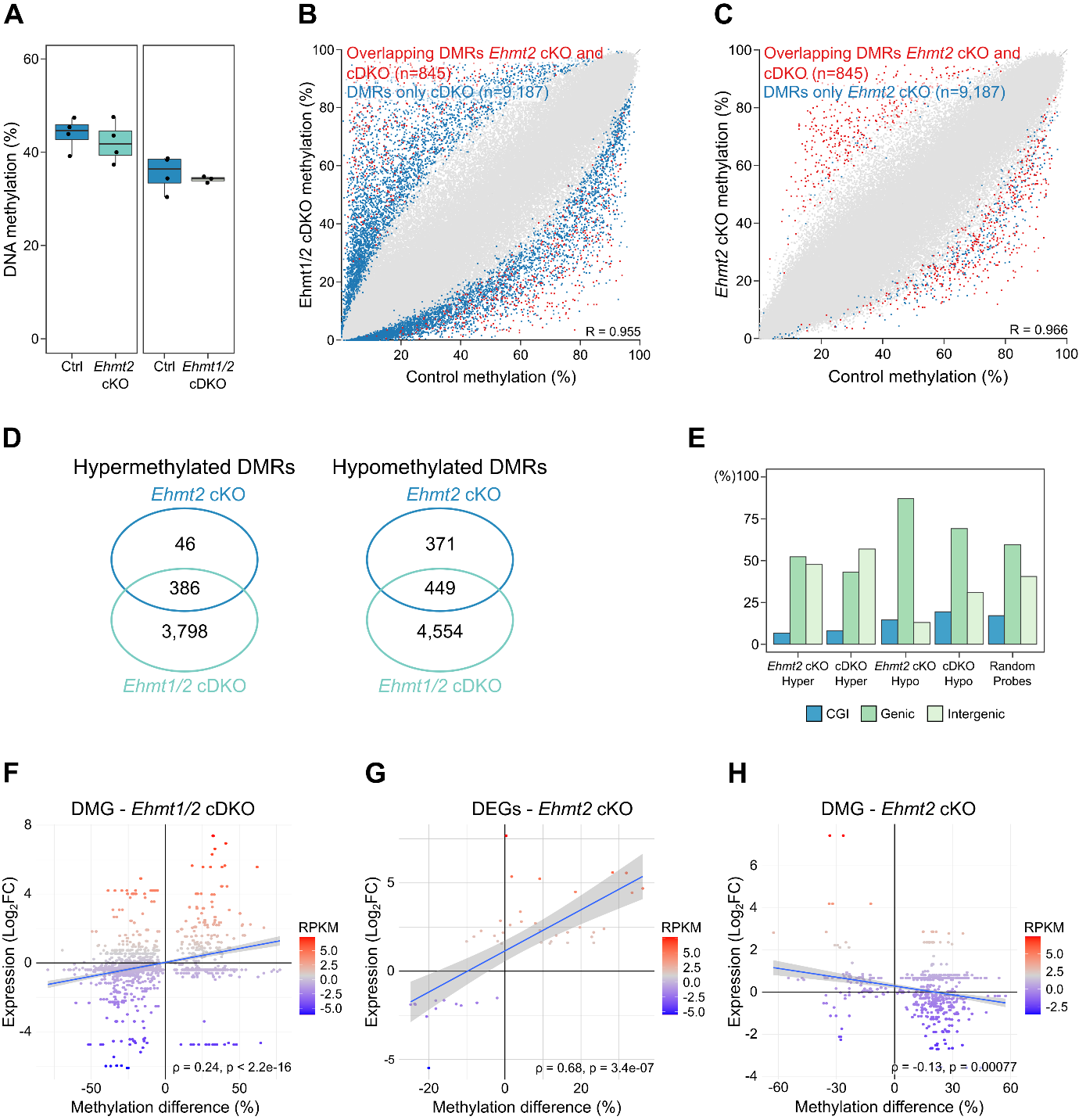


**Figure S6: DNA methylation analysis of *Ehmt2* cKO and *Ehmt1/2* cDKO oocytes with corresponding littermate controls.**

**A)** Box-whisker plots showing global DNA methylation levels using average methylation levels of 100 CpG windows. Ctrl *vs* *Ehmt2* cKO *P* = 0.5378; Ctrl vs *Ehmt1/2* cDKO *P* = 0.6077. Differences in average methylation between models may be explained by somatic cell contamination in the Ehmt2 Control and Ehmt2 cKO oocyte libraries. **B,C)** Scatterplot showing methylation levels of control vs *Ehmt1/2* cDKO **(B)** and control vs *Ehmt2* cKO oocytes **(C)**. Each dot represents the methylation values of a 100 CpG window. Unique DMRs are highlighted in blue and DMRs shared between the two genotypes in red. **D)** Venn diagrams, showing overlap of hypermethylated and hypomethylated DMRs between *Ehmt2* cKO and *Ehmt1/2* cDKO oocytes. **E)** Barchart showing percentage of DMRs and random probes overlapping CGIs, genic and intergenic regions. χ-square comparing genic and intergenic DMRs: Ehmt2 cKO *adj. P = 0.026**; all others *P < 0.0001****. **F-H)** Scatterplots showing link between methylation and expression changes of differentially methylated genes (DMG) in *Ehmt1/2* cDKO oocytes **(F)**, differentially expressed genes (DEGs) in *Ehmt2* cKO oocytes **(G)** and DMGs in *Ehmt2* cKO oocytes **(H)**. Expression levels are indicated by the colour scale. Spearman’s correlation indicated in plot.


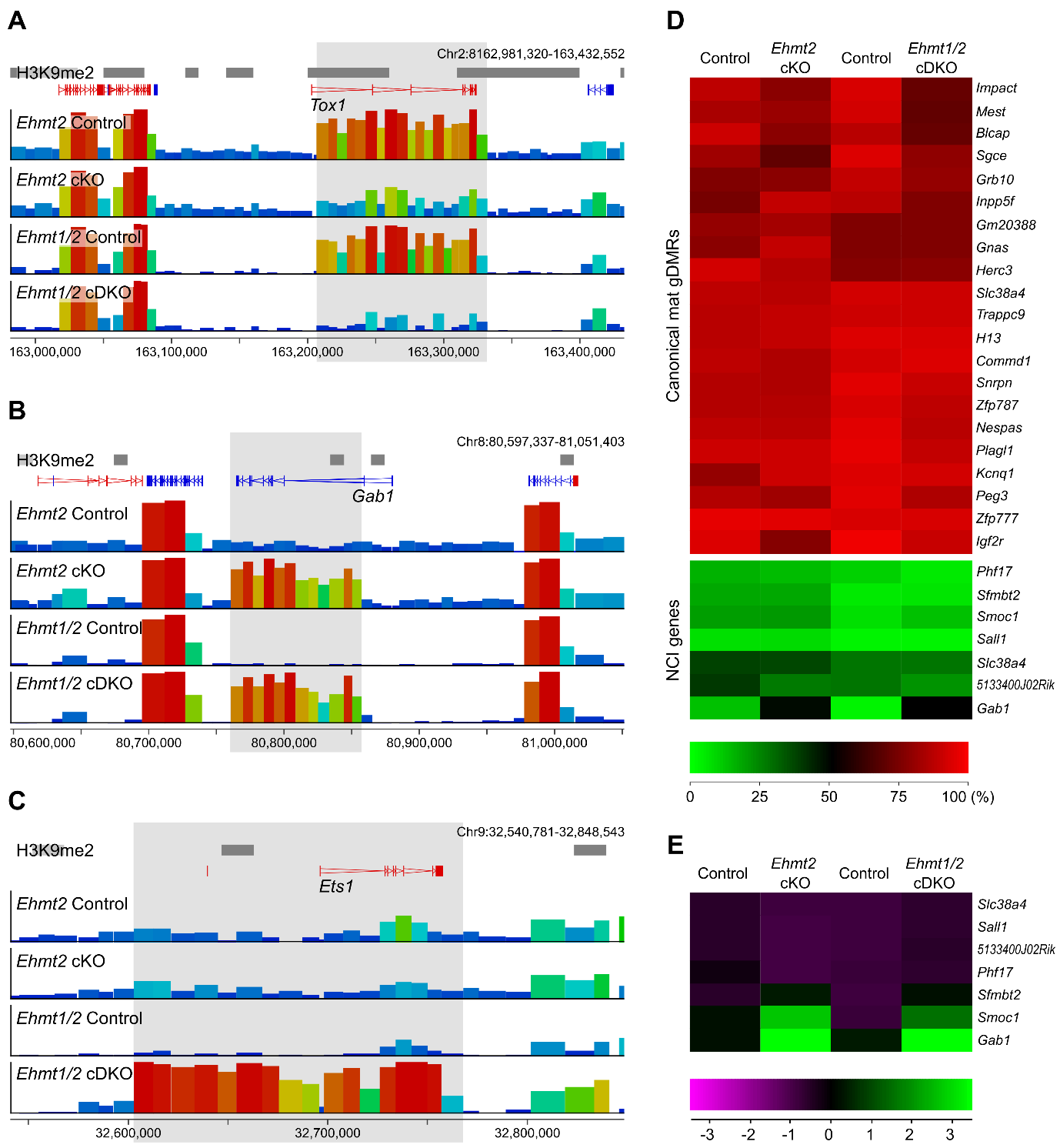


**Figure S7: Genome screenshots showing examples of differentially methylated domains and methylation and gene expression at imprinted regions.**

**A-C)** Screenshots show a region that is hypomethylated in both *Ehmt2* cKO and *Ehmt1/2* cDKO oocytes **(A)**, a region hypermethylated in both *Ehmt2* cKO and *Ehmt1/2* cDKO oocytes **(B),** and a region uniquely hypermethylated in *Ehmt1/2* cDKO oocytes **(C)**. **D)** Heatmap showing methylation levels of oocytes at maternal germline DMRs and NCI genes. **E)** Relative gene expression levels of NCI genes.


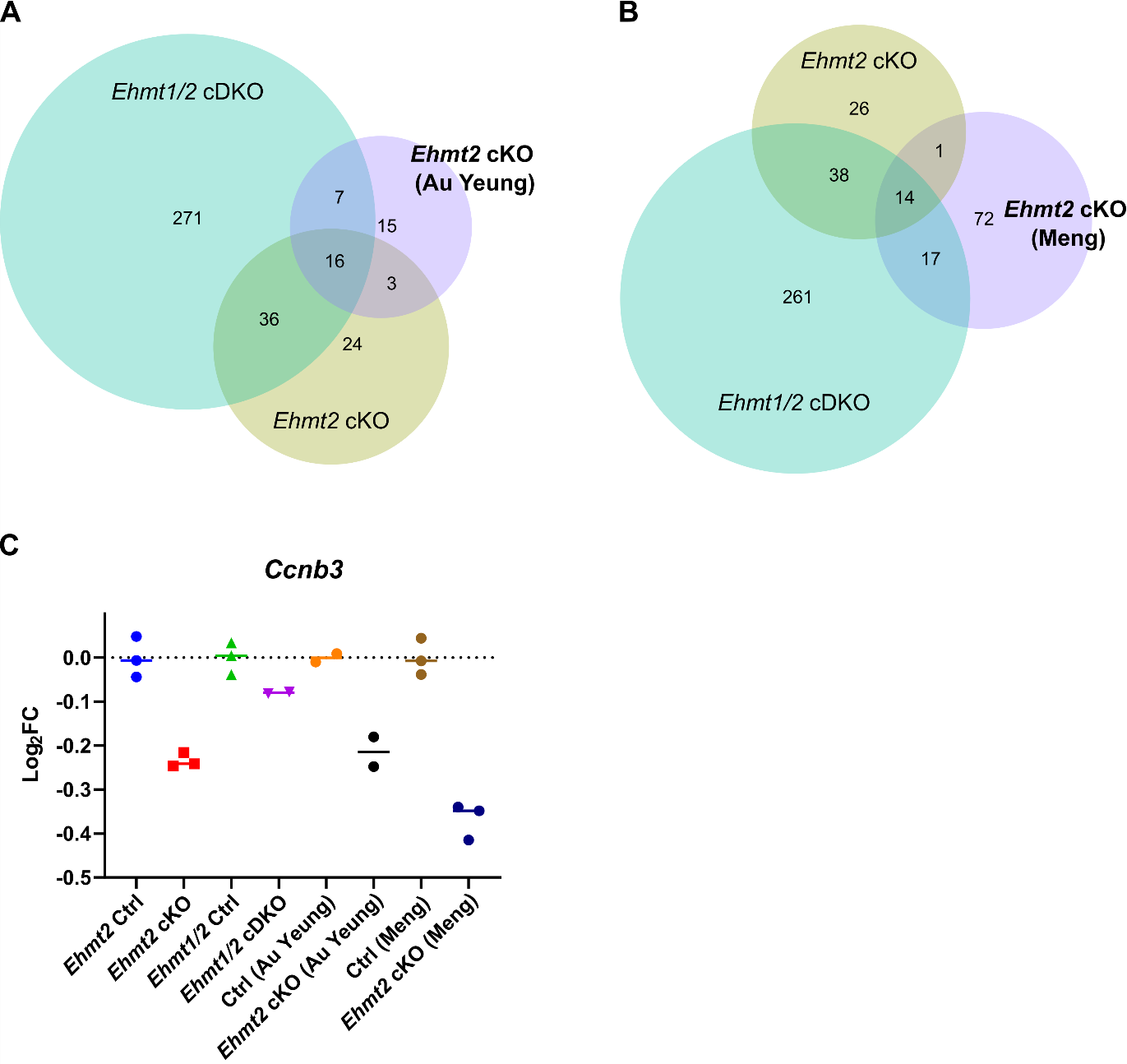


**Figure S8: Comparison of transcriptional changes between our *Ehmt2* cKO and *Ehmt1/2* cDKO models and *Ehmt2* cKO models published by Au Yeung et al. 2019 and Meng et al. 2022.**

**A, B)** Venn diagrams showing overlap of DEGs of our KO oocytes with **A)** *Ehmt2* cKO *Zp3*-Cre GV oocytes (Au Yeung) and **B)** *Ehmt2* cKO *Gdf9*-Cre MII oocytes (Meng). **C)** Log_2_FC of *Ccnb3* expression in KO oocytes compared to corresponding controls.
